# The Complete Genome of the “Flavescence Dorée” Phytoplasma Reveals Characteristics of Low Genome Plasticity

**DOI:** 10.3390/biology11070953

**Published:** 2022-06-23

**Authors:** Christophe Debonneville, Léa Mandelli, Justine Brodard, Raphaël Groux, David Roquis, Olivier Schumpp

**Affiliations:** 1 Virology, Bacteriology and Phytoplasmology, Agroscope, CH-1260 Nyon, Switzerland; lea.mandelli@agroscope.admin.ch (L.M.); justine.brodard@agroscope.admin.ch (J.B.); raphael.groux@agroscope.admin.ch (R.G.); olivier.schumpp@agroscope.admin.ch (O.S.); 2 Plant Genome Dynamics, Agroscope, CH-1260 Nyon, Switzerland; david.roquis@agroscope.admin.ch

**Keywords:** genome sequencing, effectors, “Flavescence dorée” phytoplasma

## Abstract

**Simple Summary:**

Phytoplasmas are non-cultivable organisms for which biological and molecular characterization is a challenge. Despite the development of new sequencing technologies, few high- quality phytoplasma genomes are available. This research describes the genome sequencing of the phytoplasma involved in grapevine Flavescence dorée, a devastating disease in European vineyards. Analysis of the gene content and comparison with other representative phytoplasmas revealed specific characteristics of the “Flavescence dorée” phytoplasma chromosome. Their implications for the development of the disease are presented and discussed.

**Abstract:**

Members of the genus ‘*Candidatus* Phytoplasma’ are obligate intracellular bacteria restricted to phloem sieve elements and are able to colonize several tissues and the hemolymph in their insect vectors. The current unfeasibility of axenic culture and the low complexity of genomic sequences are obstacles in assembling complete chromosomes. Here, a method combining pathogen DNA enrichment from infected insects and dual deep-sequencing technologies was used to obtain the complete genome of a phytoplasma causing Grapevine Flavescence dorée. The de novo assembly generated a circular chromosome of 654,223 bp containing 506 protein-coding genes. Quality assessment of the draft showed a high degree of completeness. Comparative analysis with other phytoplasmas revealed the absence of potential mobile units and a reduced amount of putative phage-derived segments, suggesting a low genome plasticity. Phylogenetic analyses identified *Candidatus* Phytoplasma ziziphi as the closest fully sequenced relative. The “Flavescence dorée” phytoplasma strain CH genome also encoded for several putative effector proteins potentially playing a role in pathogen virulence. The availability of this genome provides the basis for the study of the pathogenicity mechanisms and evolution of the Flavescence dorée phytoplasma.

## 1. Introduction

Phytoplasma diseases affect hundreds of plant species through highly specialized interactions whereby each phytoplasma species colonizes a relatively narrow host range [1,2,3,4]. As intracellular parasites exclusively restricted to the phloem of their host plant, they are transmitted in a persistent and propagative manner by hemipterans vectors. This mode of transmission involves multiplication in insect tissues and the crossing of several cell barriers [1,5]. Thus, the interaction with the vector is the result of advanced co-evolution [6,7]. This very high level of specialization for a limited number of hosts masks a strong capacity to adapt to changing environments such as plant phloem or insect tissues [8]. The genes required in these diverse environments are different and significant transcriptomic variation accompanies the transition from plant to vector [9,10].

Because of their small size and lack of a cell wall, phytoplasmas have been associated with organisms of the class Mollicutes since their discovery [11]. However, they are distinguished from the bacteria of the other genera of this group by their inability to be grown under axenic conditions. This specification considerably complicates their characterization and identification. Nevertheless, classification based on the 16S rRNA has led to the description of more than 30 different groups [12,13,14] including 49 officially published species and several putative species. The impossibility to cultivate them and a GC content of around 20–30% explain why, despite their high number and diversity, only 11 complete and annotated phytoplasma genome sequences are available to date.

These genomes are small (570 to 1000 kbp) and are sometimes associated with plasmids. They can have a high number of repeats and mobile units covering a significant part of the chromosome, suggesting a high plasticity of the genome [8,15,16,17]. The number of genes is low, ranging from 475 to ~1000, and phytoplasmas notably lack several biochemical pathways normally found in bacteria (reviewed in [2]). Instead, they rely on the import of essential nutrients from their environment, as evidenced by the presence of many genes encoding transport proteins [8]. The relatively small number of metabolic genes contrasts with the presence of numerous secreted effector proteins [15,18,19,20]. Effectors decrease the plant’s defenses [21] or alter its development [20,22,23,24,25] and promote the attractiveness of the plant to insect vectors [26,27,28]. These secreted effectors possess a signal peptide on the N-terminal side that may be confused with a transmembrane domain [29]. Phytoplasma genomes also contain genuine transmembrane proteins acting as effectors, as shown by Imp, Amp or VmpA, that are involved in interactions with their plant hosts or the insect vector [30,31,32,33].

In grapevine (*Vitis* sp.), phytoplasmas from various phylogenetic groups are associated with Grapevine Yellows (GY). GY is spread worldwide in wine-growing areas, inducing considerable economic losses. Flavescence dorée, one of the most devastating GY, is caused by Flavescence dorée phytoplasma (FDp), belonging to the 16SrV taxonomic group. Although it can be transmitted by several insects, the propagation of the disease is epidemic only in the presence of the leafhopper *Scaphoideus titanus* Ball [34]. FD is classified as a quarantine disease in the EU [35]. Switzerland is an interesting area for the analysis of this disease because the application of strict measures did not permit the control of the disease south of the Alps, whereas similar measures allowed the eradication of several recent outbreaks north of the Alps.

In this study, we aimed to reconstruct a high-resolution sequence of the FDp genome. A crucial step of enrichment of bacterial DNA from infected insects allowed us to obtain a high read coverage from the deep sequencing [36]. A dual sequencing approach combining Illumina and Oxford Nanopore Technology (ONT) was used to generate a complete circular and annotated chromosome of the “Flavescence dorée” phytoplasma. An analysis of key features was performed as well as a comparison with other existing phytoplasma genomes.

## 2. Materials and Methods

### 2.1. Biological Material Source

In November 2020, 5 vines of cv. Chasselas from the outbreak in Chardonne (Vaud, Switzerland) were carefully uprooted and brought to the quarantine facilities at Agroscope. These plants previously tested positive for FDp during the summer season. After overwintering, surviving plants were tested for the presence of FDp by quantitative PCR, as previously described [37]. About 30 *Scaphoideus titanus* reared according to Ripamonti et al. [38] were encaged on a symptomatic shoot for two weeks and then transferred to the healthy *Vicia faba* variety Aguadulce for transmission during two weeks. Two weeks later, the *Vicia faba* plants were tested for the presence of FDp by quantitative PCR. One positive plant was used as feeding source for about 50 *Euscelidius variegatus* 4–5th stage nymph instars for two weeks. After a latency period of two weeks, twenty *E. variegatus* were collected for DNA extraction.

### 2.2. DNA Extraction and Enrichment

Twenty insects were homogenized in 900 μL of extraction buffer (3% Cetyltrimethylammonium bromide CTAB, 1.4 M NaCl, 25 mM EDTA, 1 M Tris-HCl, 2% β-Mercaptoethanol, pH 8.0) and shaken for 30 min at 600 rpm and 65 °C. 900 μL of Chloroform/Isoamylalcohol was added, homogenized by vortexing for 5 s and centrifuged for 5 min at 3000× *g*. The aqueous layer was carefully transferred to a new tube, mixed with an equal volume of cold Isopropanol, and incubated 60 min at −20 °C for DNA precipitation. Precipitated material was recovered by 2 min of centrifugation at 10,000× *g* and washed with 1 mL of 70% Ethanol. A DNA pellet was dried overnight at room temperature and resuspended into 100 μL of PCR-grade water. The extract was treated with 1 mg/mL RNase A for 30 min at 37 °C. A total of 2 μg of DNA was used with the NEBNext Microbiome DNA Enrichment kit (New England Biolabs Inc., Ipswich, MA, USA #E2612S), following manufacturer’s instructions. The option “Ethanol Precipitation” was applied to clean up the DNA at the end of the procedure.

### 2.3. MinION and Illumina Sequencing

DNA was fragmented using a g-tube (Covaris, Woburn, MA, USA #520079) for 1 min at 2000× *g*. A total of 1 μg of fragmented DNA was used with the Ligation Sequencing kit (Oxford Nanopore Technologies, Oxford, UK #SQK-LSK110), following manufacturer’s instructions. The final mixture containing the library was sequenced on a MinION Mk1C using a R9.4.1 flow cell. Sequences from the MinION were basecalled and adapter sequences removed with Guppy v3.4.5 using built-in configuration “dna_r9.4.1_450bps_fast.cfg”. Reads were then processed through Filtlong v0.2.0 with --min_mean_q 80 and --min_length 200 to remove low-quality sequences. The long reads were obtained from two sequencing runs of two independent libraries on the same flow cell. They had a N50 of 15.5 kb, as assessed by NanoPlot. Illumina sequencing was performed by Macrogen Europe B.V (Amsterdam, The Netherlands) using TruSeq DNA PCR-Free library preparation, followed by a NovaSeq 2 × 150 bp sequencing run and a depth of 100 M reads. Reads were trimmed through trimmomatic v0.39 with PE –phred33 ILLUMINACLIP LEADING:20 TRAILING:20 SLIDINGWINDOW:4:20 MINLEN:50.

### 2.4. De Novo Genome Assembly and Quality Assessment

Long reads were assembled with Flye v2.8.3-b1695. All resulting contigs were blasted against a local database (built with makeblastdb application) of phytoplasma complete genomes with Blastn v2.5.0+. Long and short reads were mapped using Minimap2 v2.21-r1071 and Bowtie2 v2.3.4.1, respectively. Mapped reads were processed together for hybrid assembly using Unicycler v0.4.9b. Additionally, mapped reads were visually inspected using Geneious v2022.0.2 for polishing of possible assembly errors based on MinION long reads stackings, similar to Huang et al., 2022 [16]. Samtools depth function was used to assess the sequencing coverage of both long and short reads. Completeness of the draft genome was estimated using BUSCO v5.3.0 [39], with the lineage dataset “mollicutes_odb10” created on 6 March 2020. Additionally, the webserver MiGa (http://microbial-genomes.org, accessed on 20 June 2022) [40] was used for taxonomic evaluation. Sequences were deposited in GenBank database under accession number CP097583 for the genome and PRJNA838420 for the Sequence Read Archive (SRA).

### 2.5. Functional Annotation

The final draft was annotated with Prokka v1.13 [41]. Functional characterization was assessed with GO FEAT (http://computationalbiology.ufpa.br/gofeat/, accessed on 20 June 2022) [42] and BlastKOALA (https://www.kegg.jp/blastkoala/, accessed on 20 June 2022) [43]. Additionally, manual curation was performed by BlastP searches against the NCBI non-redundant database. Putative effector proteins, including those with transmembrane domain, were predicted with SignalP v5.0 [44] and Phobius (https://phobius.sbc.su.se/, accessed on 20 June 2022) [45]. The circular map was generated using Circlize v0.4.10 [46]. The first base of the start codon of the dnaA gene was set as base pair 1.

### 2.6. Genome Comparison and Phylogenetic Analyses

For comparative analysis with representative phytoplasma genomes, OrthoVenn2 (https://orthovenn2.bioinfotoolkits.net/home, accessed on 20 June 2022) [47] was used to identify orthologous clusters. Multiple sequence alignments of single-copy orthologue groups were prepared with MUSCLE v3.8.1551 and concatenated to produce one super-matrix for each phytoplasma. Maximum likelihood phylogeny was inferred with IQtree (http://iqtree.cibiv.univie.ac.at/, accessed on 20 June 2022) [48] with substitution model set on “auto” and bootstrapping with 1000 replicates. For groEL and smpB, trees were inferred by maximum likelihood method in MEGA using the General Time Reversible model and bootstrapping with 500 replicates. All trees were visualized with iTOL (https://itol.embl.de/, accessed on 20 June 2022) [49].

## 3. Results

### 3.1. Genome Assembly and Quality Assessment

**Figure 1:**
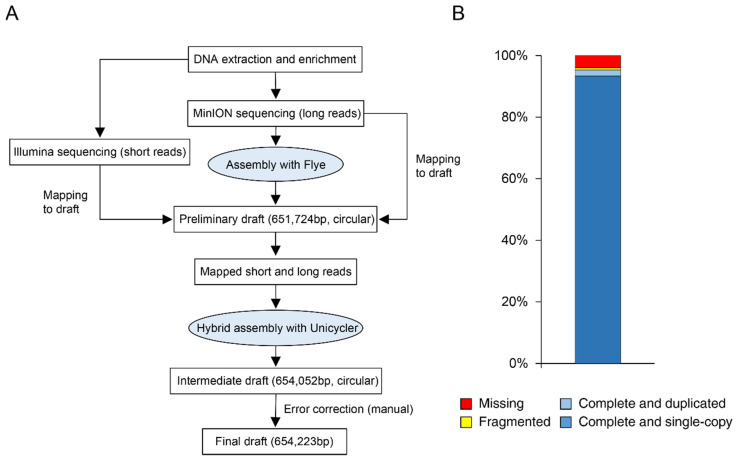
Genome assembly and quality assessment. (**A**) Workflow proposed as the successful approach for de novo genome assembly. (**B**) BUSCO analysis to assess completeness of the assembled chromosome.

Twenty *Euscelidius variegatus* fed on FDp-infected broad beans were used as starting material. DNA extract was enriched in phytoplasma genomic DNA through a methyl-CpG-binding, domain-mediated method [36]. Quantitative PCR analysis based on map locus produced a reduced Cq value of roughly five cycles (data not shown) corresponding to a 30-times enrichment. To improve sequencing quality, we used both long-read sequencing from MinION nanopore and short-read sequencing from Illumina. MinION nanopore sequencing generated a total of 1,158,896 long reads (representing 7.3 × 10^9^ bp), and Illumina sequencing generated 128,906,832 short reads (representing 19.5 × 10^9^ bp). In the first step of de novo assembly, long reads were assembled with “Flye”, resulting in 10,820 contigs. A comparison with a phytoplasma genome database by MegaBlast identified 65 phytoplasma-related contigs. Among them, a circular contig of 651,724 bp (Figure 1A) showed a bitscore of 4.3 × 10^4^ with the genome of *Candidatus* Phytoplasma ziziphi, a phytoplasma belonging to 16Sr V group [50]. The remaining contigs showed only a poor match with phytoplasma sequences (Supplementary Table S1), and no plasmids were detected. In the second step, all long and short reads were mapped to this contig and only mapped reads were used as inputs for a hybrid assembly with “Unicycler”, resulting in a circular chromosome of 654,052 bp. Finally, the reads mapped to chromosome were visually inspected and errors introduced by the long reads corrected at base-pair level to obtain the final assembly of 654,223 bp. Mapping results to this draft found a 15× and 46× mean coverage for long and short reads, respectively.

BUSCO analysis evaluated 151 markers from the dataset “Mollicutes”, resulting in 143 genes considered as complete (either single or duplicated), one fragmented gene, and seven missing genes (94.7%, 0.7%, and 4.6% respectively; Figure 1B). In comparison, the assessment of the published genome of *Candidatus* Phytoplasma ziziphi showed a lower BUSCO score (data not shown). Analysis with MiGa [40], an online tool to assess prokaryotic genome assemblies, rated the completeness as “Very high”, further confirming the quality of the circular chromosome.

### 3.2. Genome Characteristics and Annotation

#### 3.2.1. General Features

**Figure 2:**
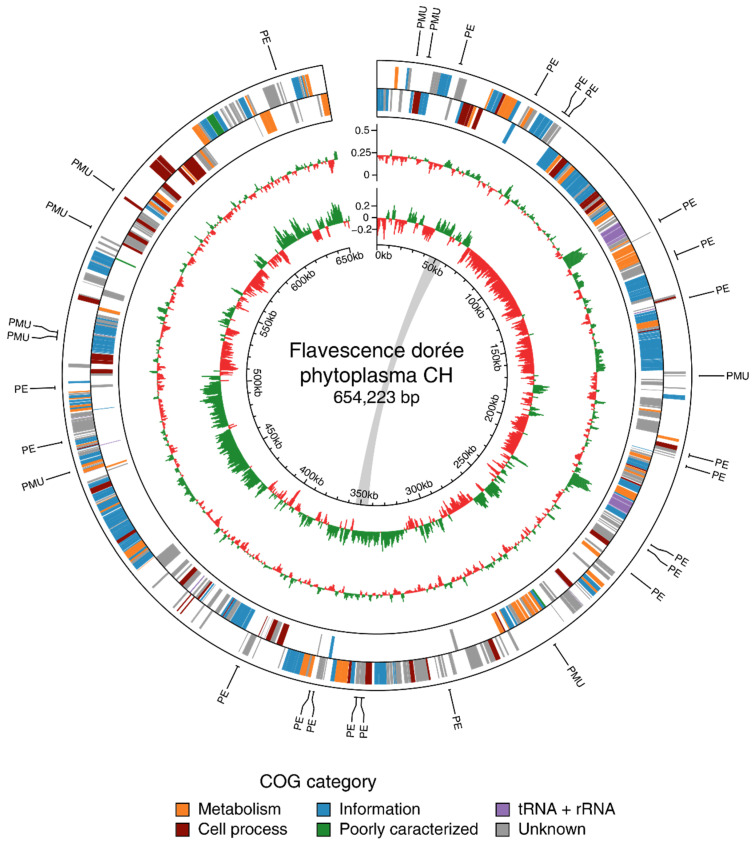
Genome map of the circular chromosome of FDp strain CH. Rings from outside in: (1 and 2) coding sequences on the forward and reverse strand, respectively (color-coded by functional categories); (3) G/C content (above average: green; below average: red); (4) G/C skew (above average: green; below average: red); (5) scale marks. (PE): putative effectors; (PMU): genes belonging to the potential mobile unit core set. The grey line indicates the positions of the 10 kb duplicated region. The G/C content and G/C skew are displayed with a sliding window of 1 kb and a step size of 0.5 kb.

The genome of Flavescence dorée phytoplasma consists in a circular chromosome of 654,223 bp with a G/C content of 21.7%, which places it among the species with the lowest GC rate [51]. The sequence shows a regular GC-skew pattern comparable to ‘*Ca*. P. mali’ chromosome (Figure 2) [52]. The annotation revealed 506 protein-coding genes (CDS), 32 tRNA genes, and 2 rRNA operons (Table 1, Figure 2). CDSs have an average length of 987 bp and cover 77% of the total size of the chromosome. Among the CDSs, 350 proteins had an assigned function, whereas 156 were categorized as hypothetical products. A screen of protein databases searching for homology revealed a match for 470 of the 506 annotated proteins (with an e-value < 1 × 10^−10^). Proteins with a homologous peer in the genus *Candidatus* Phytoplasma represented 90.9% of the total.

#### 3.2.2. Functional Annotation

**Figure 3:**
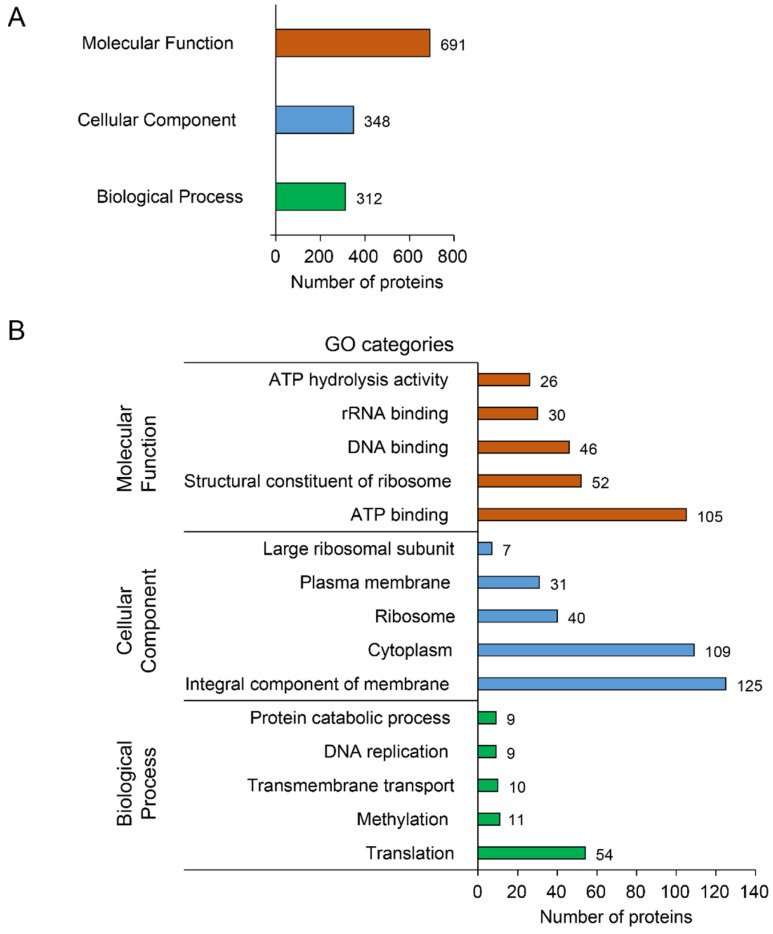
Gene Ontology categories associated with the CDSs from FDp strain CH genome. (**A**) Bar chart showing the distribution among the three main GO domains. One protein can have several molecular functions. (**B**) Bar chart showing the five most represented GO categories in each domain.

**Figure 4:**
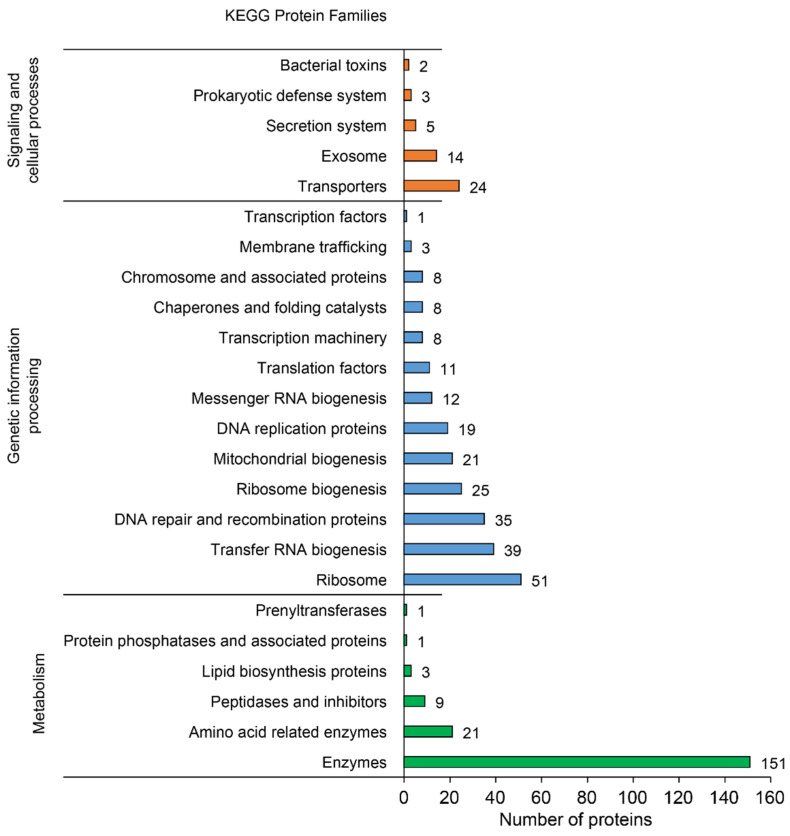
Bar chart representing the KEGG protein families associated with the CDSs from FDp strain CH genome.

Proteins associated to a Gene Ontology (GO) term were classified into the three general categories: “Biological Process” (312 hits), “Cellular Component” (348 hits), and “Molecular Function” (691 hits) (Figure 3A). The most abundant biological process was “Translation” (54 hits), followed by “Methylation” (11 hits) and “Transmembrane transport” (10 hits). In the cellular component category, the major part of the proteins belonged to “Integral component of membrane” (125 hits), “Cytoplasm” (109 hits), and “Ribosome” (40 hits). Finally, the most abundant molecular functions were “ATP binding” (105 hits), “Structural constituent of ribosome” (52 hits), and “DNA binding” (46 hits) (Figure 3B and Supplementary Table S2). Functional annotation with BlastKOALA (with reference taxonomy group NCBI:txid33926 = Tenericutes) resulted in the assignment of 320 of the 506 CDSs (63.2%) to orthologues in the KEGG database (prokaryotes) [53]. The three main protein families contained 184 proteins classified in “Metabolism”, 241 in “Genetic information processing”, and 48 in “Signaling and cellular processes” (Figure 4).

### 3.3. Key Features

#### 3.3.1. Transporters and Metabolic Genes

As shown for other phytoplasmas, FDp has reduced metabolic functions with several incomplete pathways, such as, for example, those for fatty and amino acids biosynthesis. Instead, phytoplasmas depend on the import of essential elements, and the present genome possessed 40 genes involved in transport, most of them being ABC transporters (25 genes; Supplementary Table S3). The pathogen had a complete protein secretion system (sec) with 10 genes affiliated with this function (secA, secE, secY, yidC, ffh, ftsY, dnaJ, dnaK, grpE, and groL) [54]. A total of 25 genes, including 16 coding for ATP-dependent zinc metalloproteases (FtsH proteins), were associated with a peptidase activity. As for the carbohydrate metabolism, the chromosome encoded for complete glycolysis and pyruvate oxidation pathways (Supplementary Table S2). Notably, the genome contained six genes with a restriction endonuclease function, a system that does not belong to the shared core set of phytoplasmas.

#### 3.3.2. Effector Genes and Potential Mobile Units

SignalP v5.0 [44] and Phobius [45] were used to predict the presence of signal peptide (SP) in the set of proteins. A total of 17 putative secreted proteins were identified (Table 2). Phobius was designed to better separate transmembrane domains and signal peptides [29]. It predicted a transmembrane domain instead of a signal peptide for three of these proteins (FlDop_00101, FlDop_00265 and FlDop_00298) but also suggested an SP in FlDop_00246 and FlDop_00266, whereas SignalP v5.0 did not detect them (Supplementary Figure S1). Interestingly, Phobius predicted both a transmembrane domain and an SP for three proteins (FlDop_00023, FlDop_00048, FlDop_00049). These proteins may act as pathogenicity factors, with different mechanisms either attached to the membrane or released in the host’s cells. Only one homologue of a member of the SAP repertoire describing putative effector proteins identified in ‘*Ca*. P. asteris’ [18] was present (FlDop_00531), and no homologues to the experimentally characterized effectors SAP05, SAP11, SAP54 and TENGU were found.

Five proteins defined as “hypothetical transmembrane protein (htmp)” were also considered as potential effectors. In addition, FtsH proteins mentioned above can also act as potential virulence agents, as shown for ‘*Ca*. P. mali’ [55] and hypothesized for FDp [10].

Phytoplasma genomes usually contain regions called Potential Mobile Units (PMUs) derived from ancient phage attacks [56,57]. PMUs identified in various phytoplasma species present a set of conserved genes involved in different functions, such as transposition and gene duplication [8,16]. Most described PMUs also harbor putative effector proteins. Interestingly, no PMU-like region had been identified in the genome of FDp. Although genes belonging to the core set of PMUs were present (Supplementary Table S2), they were scattered over the circular genome and were only single copy (Figure 2). A raw estimation of prophage loci with PHASTER [58] identified one incomplete region of about 16 kb (2.5% of the total chromosome size) of putative viral origin.

Another interesting finding was the presence of a duplicated region with no detectable phage-derived characteristics of about 10 kb at positions 43,623 to 54,237 and 344,306 to 354,721 containing eight genes. Most of them were involved in sugar transport and translation processes. The region showed a good synteny with several phytoplasmas (data not shown).

### 3.4. Phylogeny and Taxonomy

**Figure 5:**
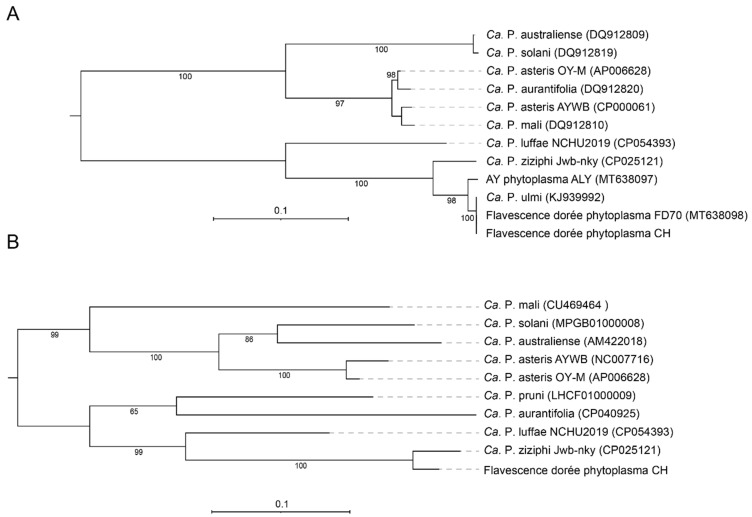
Maximum likelihood phylogeny based on nucleotide sequences of (**A**) groEL and (**B**) smpB genes. The numbers on branches indicate the level of bootstrap support (500 replicates). Support values above 65% are labeled. The scale bar shows the number of substitutions per site.

According to the current classification system based on the 16S rRNA gene [59,60], FDp belongs to group 16SrV. Both 16S rRNA gene copies found in the present genome had 100% identity with the gene from reference isolate FD92, placing the strain in the subgroup V-D and cluster map-FD2 [61]. In agreement, the sequence of the Methionine aminopeptidase (map) gene was 100% identical to the described genotype M54. The phylogenetic tree constructed with DNA sequences from the intracellular chaperone groEL (Figure 5A) grouped the new strain together with *Candidatus* Phytoplasma ulmi, Aster Yellows Phytoplasma, and *Candidatus* Phytoplasma ziziphi, three members of the 16SrV group. An additional tree based on smpB, a gene coding for a protein involved in ribosomal rescue recently shown to resolve species differentiation in *Acholeplasmataceae* [62], placed *Candidatus* Phytoplasma ziziphi as the closest relative of the new strain (Figure 5B). At the whole-genome level, these two phytoplasmas have an average nucleotide identity (ANI) of 92.2%, therefore likely representing distinct species [63]. Analysis with MiGa confirmed that the circular chromosome most likely belonged to a species not present in the database (with *p*-value = 0.0025).

### 3.5. Full Genome Comparison

**Figure 6:**
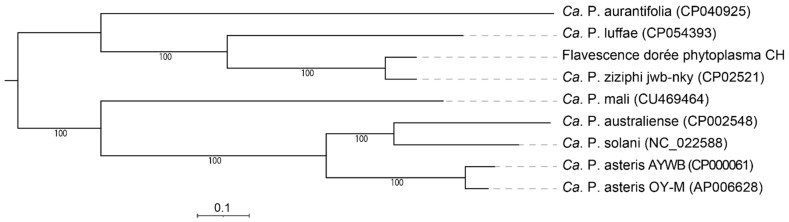
Maximum likelihood phylogeny inferred using 203 single-copy orthologue genes; the concatenated alignment contains 70,619 aligned amino acid sites. The numbers on branches indicate the level of bootstrap support (1000 replicates). Support values above 80% are labeled. The scale bar shows the number of substitutions per site.

Comparative analysis of gene contents between FDp and eight other phytoplasma genomes (phytoplasmas listed in Table 1 and ‘*Ca*. P. solani’) identified 227 orthologous clusters shared by all phytoplasmas analyzed, including 203 single-copy gene clusters with one protein for each species. The phylogenetic tree based on the concatenated alignment of these proteins confirmed the phylogeny built on smpB, with ‘*Ca*. P. ziziphi’ being the nearest member and ‘*Ca*. P. australiense’ the most distant one (Figure 6). Moreover, FDp had an average amino acid identity (AAI) of 88.4% and 57.7% when compared to ‘*Ca*. P. ziziphi’ and ‘*Ca*. P. australiense’, respectively.

Pairwise comparisons showed that FDp and ‘*Ca*. P. ziziphi’ shared the highest number of clusters (Table 3), further corroborating the two phylogenetic analyses. Seven clusters totaling 13 proteins with unknown function were found exclusively in FDp as well as 56 “singletons” (i.e, proteins) with no homologues in other phytoplasma genomes (Supplementary Table S4). The majority of these singletons were hypothetical proteins (46). Notably, seven of them were “putative effectors” and two were associated with a restriction enzyme activity.

## 4. Discussion

Despite the small sizes of their genomes, phytoplasmas are challenging organisms to sequence. Because of the low complexity of their chromosomes and the impossibility of axenic culture leading to subsequent excess of host DNA, de novo assemblies often contain unresolved gaps and incomplete genome sequences. The source material for DNA extract is also critical to optimize the abundance of phytoplasma DNA. Thus, various approaches to increase the proportion of pathogen DNA in the extract or the combination of complementary sequencing techniques were recently applied to reconstruct the full genome of several phytoplasmas [36,64]. The “Flavescence dorée” phytoplasma is not an exception and resisted sequencing efforts for 10 years since the publication of a short communication on a partial assembly in 2011 [65]. Different strategies were tested in our laboratory to achieve whole genome assembly. Sequencing of infected plants with or without Rolling Circle Amplification (RCA) enrichment resulted in numerous gaps and contigs, covering up to a maximum of 80% of the current chromosome. Finally, the successful approach relied on DNA extraction from infected *Euscelidius variegatus* with an enrichment step based on depletion of methylated DNA followed by the combination of both Illumina and ONT sequencing platforms. Using the produced reads, a hybrid strategy with Unicycler led to the assembly of a ~654 kb circular chromosome. Concordantly, the estimated size of the FD92 strain deduced by restriction digests and Pulse-field Gel Electrophoresis (PFGE) is ~670 kb [66].

Quality assessment of the draft genome with BUSCO showed that the sequence encodes for more than 95% of the core genes from the database “Mollicutes” (144/151). Among the seven missing genes, two are actually present (GTPase Era, energy-coupling factor ATPase) but not detected by the software, despite their strong identity with homologues in other phytoplasmas. Interestingly, three genes (Ribose-phosphate pyrophosphokinase, Uracil phosphoribosyltransferase, and Glutamine N5 methyltransferase) are also absent from the phytoplasma genomes listed in Table 1. The gene encoding for Cytidylate kinase is only missing in ‘*Ca*. P. ziziphi’ and ‘*Ca*. P. luffae’, the two phytoplasmas most closely related to FDp. Lastly, Ribonuclease R has been shown to be present in Acholeplasmas but replaced by an inorganic phosphatase in all phytoplasmas [62].

Functional classification of annotated genes revealed that those assigned to KEGG categories “Genetic information processing” (transcription, translation, replication) and “Metabolism” were the most represented. This result is in accordance with previous phytoplasma genome analyses [15,16,67]. A complete set of genes related to glycolysis, either from the upper part (energy demanding) or from the lower part (energy yielding), is present in the genome of FDp, indicating this pathway as the major source of energy. This was already suggested for other phytoplasmas [68,69]. The source of sugars could be sucrose and trehalose, the two major sugar types in plant phloem and insect haemolymph, respectively [70,71]. Maltose ABC-type transporters have been proposed to import these sugars into the cytoplasm [67,68]. An alternative pathway with malate as a carbon source for ATP synthesis is plausible as well. Indeed, three malate/citrate symporters, a malate dehydrogenase, and a phosphate propanoyl transferase were identified in the genome, and malate has been proposed to be a predominant organic acid in the phloem sap [72]. Interestingly, four genes involved in sugar transport are present in the 10 kb duplicated region (2 permeases and 2 substrate-binding proteins). These proteins are members of the Ugp (UgpB1 and UgpC1) and Mal (MalF and MalG) transport systems, which have been shown to be exchangeable in *Escherichia coli* to form a functional periplasmic permease [73]. In addition, a recent study highlighted that the ugp-based system was essential for *Mycobacterium tuberculosis* pathogenicity in humans [74], suggesting that the duplication of this locus in FDp may have a relevance in pathogenicity and virulence of the phytoplasma.

Like other phytoplasmas, FDp harbors a complete sec secretion system to export effectors into host cells [75]. Based on the prediction of signal peptide by SignalP v5.0 and Phobius, 17 putative secreted proteins were identified, among which 14 are devoid of a transmembrane domain and 7 are unique to FDp. This number of predicted effectors is surprisingly low. In comparison, other phytoplasmas (e.g., ‘*Ca*. P. asteris’, ‘*Ca*. P. ziziphi’) usually contain up to five times more candidates in this category [18,50]. Fourteen of them encode a hypothetical protein and only one is a SAP-like protein [18]. Notably, no homologues of SAP05, SAP11, SAP54, and TENGU were found. These proteins produce important morphology changes such as phyllody- or witches’ broom-like symptoms in plants infected with various phytoplasmas [23,25,76,77,78,79]. Accordingly, vines infected with FDp do not present these types of symptoms. Of interest are also the five genes annotated as hypothetical transmembrane proteins (Htmps) identified in the “Flavescence dorée” chromosome that may mediate interactions with their host environment. Indeed, VmpA mediates interaction within the midgut and salivary glands of the insect vector [30,80] and Imp interacts with plant actin [31] as well as protein extracts from several insects [81]. Future work is needed to characterize these potential pathogenic agents and their roles in the development of the disease cycle in plant and/or insect hosts.

Putative transposon-like structures named PMUs are unique to phytoplasmas. It has been suggested that they contribute to the genome plasticity of these bacteria [8,82] and are found in the 19 genome assemblies analyzed by Huang and coworkers [16]. In some species, these PMUs account for up to 25% of the genome [16,83]. These regions play a key role in the variance of genome sizes among phytoplasmas. Strikingly, no PMUs were identified on the chromosome of FDp, confirming preliminary results from Carle and colleagues [65]. Although some core PMU genes are present in the sequence, they are scattered throughout the chromosome. Genome instability can also be the consequence of ancient phage integration. Wei et al. [57] determined that large portions of the genome of ‘*Ca*. P. asteris’ strains OY-M and AYWB represented cryptic phage-derived genes (31% and 22.7% respectively). A Prophage Finder tool identified one partial region of putative phage origin in the chromosome of FDp representing less than 2.5% of the total size. Among the 105 proteins with an ATP binding function, sixteen genes encode for FtsH (or HflB) proteins. In *E. coli*, HflB proteins have been shown to prevent prophage integration into the bacterial chromosome by efficient degradation of the λ CII regulatory protein [84]. Moreover, several genes with restriction enzyme activity are predicted in the chromosome and may have played a role in protecting the genome from an integration event following phage infection. However, restriction enzyme activities do not belong to the core set of phytoplasmas [68]. Taken together, these findings suggest that the Flavescence dorée phytoplasma is well armed against phage attacks and is possibly less prone to gene rearrangement than other genomes of the lineage. A regular GC skew pattern (Figure S2) also supports this hypothesis. Remarkably, the genome of ‘*Ca*. P. solani’, a phytoplasma producing the same symptoms in grapevine, shows a completely different layout with a high degree of plasticity, as evidenced by large genome size, numerous gene duplications, and irregular GC skew [67]. It is hypothesized that the genome plasticity favors the spread by multiple insect vectors and infection of a larger diversity of host plants. Small genome size of phytoplasmas is considered to be a consequence of the reductive evolution favored by high intracellular specialization. However, this process is suspected to be balanced by genome plasticity mostly driven by PMUs, leading to gene duplications. Mechanisms by which FDp has evolved towards a more stable genome structure are yet to be determined. Nevertheless, highly specialized interactions restricted to the *Vitis* sp.-*S. titanus*-FDp pathosystem are thought to be responsible for a rapid evolution of the phytoplasma following the introduction of *Scaphoideus titanus* in Europe, presumably in the early 1900s [6]. The reduced number of effectors identified in FDp and the absence of the most common effectors, which are SAP05, SAP11, SAP54, and TENGU, support this hypothesis.

## 5. Conclusions

This study provides the assembly of the “Flavescence dorée” phytoplasma complete genome. Comparative analysis demonstrated the existence of conserved features among phytoplasmas as well as specific characteristics of the Flavescence dorée pathogen. A significant finding was that the genome did not contain a PMU-like region and appeared to be poor in rearrangement events. Of particular interest would be the functional study of putative effector genes for a better understanding of the interactions between the pathogen and its hosts. Furthermore, future studies of closely related strains detected on *Alnus* sp., presumed to be the ancestor population of FDp [61], will be essential to reconstruct the lineage of Ca. Phytoplasma sp. causing Grapevine Yellows.

## Figures and Tables

**Table 1:** General characteristics of FDp strain CH and comparison to representative complete phytoplasma genomes.

Strain (Accession)	16S rRNA Group	Genome Size (bp)	G/C Content (%)	No. of CDS	Protein-Coding Regions (%)	No. of tRNA Genes	No. of rRNA Operons
FDp strain CH (CP097583)	V	654,223	21.7	506	77	32	2
‘*Ca*. P. ziziphi’ jwb-nky (CP02521)	V	750,803	23.2	671	78	32	2
‘*Ca*. P. asteris’ AYWB (CP000061)	I	706,569	26.9	636	73.9	32	2
‘*Ca*. P. asteris’ OY-M (AP006628)	I	853,092	27.8	752	72.8	32	2
‘*Ca*. P. luffae’ (CP054393)	VIII	769,143	23.3	725	80.1	31	2
‘*Ca*. P. australiense’ (CP002548)	XII	959,779	27.2	928	75.5	35	2
‘*Ca*. P. mali’ (CU469464)	X	601,943	21.4	500	78.2	32	2
‘*Ca*. P. aurantifolia’ (CP040925)	II	635,584	24.5	471	66.1	24	2

**Table 2:** Putative effectors encoded by the FDp genome.

Protein ID	Function	SignalP5.0	Phobius	Accession # of Orthologues in Other Phytoplasmas (Max. 2)
FlDop_00023	Variable membrane protein B	SP	SP and 1 TM	VIO49504 (Alder yellows P.)
FlDop_00041	Hypothetical protein (htmp2)	no prediction	TM	WP_225696264 (*Ca*. P. sp. AldY-WA1) WP_121463947 (*Ca.* P. ziziphi)
FlDop_00048 *	Hypothetical protein	SP	SP and 1 TM	none
FlDop_00049 *	Hypothetical protein	SP	SP and 1 TM	none
FlDop_00090	Hypothetical protein	SP	SP	WP_225696128 (*Ca*. P. sp. AldY-WA1) WP_121464024 (*Ca.* P. ziziphi)
FlDop_00101	Hypothetical protein	SP	TM	WP_238055118 (*Ca*. P. ziziphi) WP_121464035 (*Ca.* P. ziziphi)
FlDop_00112	Hypothetical protein	SP	SP	AYJ01330 (*Ca.* P. ziziphi)
FlDop_00153	Hypothetical protein (htmp5)	no prediction	TM	WP_121464226 (*Ca*. P. ziziphi)
FlDop_00158 *	Hypothetical protein	SP	SP	none
FlDop_00183	Hypothetical protein	SP	SP	WP_026072021 (Poinsettia branch-inducing P.) WP_152411650 (Milkweed yellows P.)
FlDop_00185	Hypothetical protein (SVM family)	SP	SP	WP_034172411 (Chrysanthemum yellows P.) WP_024563506 (*Ca*. P. tritici)
FlDop_00190	Hypothetical protein	SP	SP	PQP79517 (*Ca*. P. phoenicium) WP_078123062 (Ca. P. aurantifolia)
FlDop_00246 *	Hypothetical protein	no prediction	SP	none
FlDop_00265	Hypothetical protein	SP	TM	WP_121464113 (*Ca*. P. ziziphi)
FlDop_00266	Hypothetical protein	no prediction	SP	WP_121464114 (*Ca*. P. ziziphi)
FlDop_00278 *	Hypothetical protein	SP	SP	none
FlDop_00279 *	Hypothetical protein (htmp1)	no prediction	TM	none
FlDop_00298	Hypothetical protein	SP	TM	WP_012504569 (*Ca*. P. mali) WP_227807101 (Mulberry dwarf P.)
FlDop_00404	Hypothetical protein (htmp3)	no prediction	TM	WP_225696004 (*Ca*. P. sp. AldY-WA1) WP_121463722 (*Ca.* P. ziziphi)
FlDop_00420 *	Hypothetical protein (htmp4)	SP	SP	none
FlDop_00531	SAP21-like protein	SP	SP	QKX95099 (Rapeseed phyllody P.) WP_122225587 (*Ca*. P. solani)

SP: signal peptide; TM: transmembrane domain. * singletons (proteins with no homologue in other analyzed phytoplasma genomes).

**Table 3:** Pairwise genome comparisons.

	FDp Strain CH	‘*Ca*. P. solani’	‘*Ca*. P. asteris’ OY-M	‘*Ca*. P. asteris’ AYWB	‘*Ca*. P. ziziphi’	‘*Ca*. P. mali’	‘*Ca*. P. luffae’	‘*Ca*. P. aurantifolia’
‘*Ca*. P. australiense’ (NC_021236)	306	335	332	337	302	301	305	290
‘*Ca*. P. aurantifolia’ (CP040925)	295	275	282	291	293	295	309	
‘*Ca*. P. luffae’ (CP054393)	321	300	301	301	322	307		
‘*Ca*. P. mali’ (NC_011047)	300	299	303	301	302			
‘*Ca*. P. ziziphi’ jwb-nky (CP02521)	359	296	307	303				
‘*Ca*. P. asteris’ AYWB (NC_007716)	299	329	384					
‘*Ca*. P. asteris’ OY-M (AP006628)	302	343						
‘*Ca*. P. solani’ (GCA_000970375)	304							

The numbers indicate the orthologue clusters between any pair of genomes.

## Data Availability

Sequences were deposited in GenBank database under accession number CP097583 for the genome and PRJNA838420 for the Sequence Read Archive (SRA).

## Supplementary Materials

The following supporting information can be downloaded at: https://www.mdpi.com/article/10.3390/biology11070953/s1, Figure S1: Graphs from Phobius prediction with putative effectors; Figure S2: GC skew plots of representative phytoplasma genomes; Table S1: List of contigs from the initial assembly with Flye; Table S2: Complete list of CDSs annotation in different databases; Table S3: CDSs associated with different representative functions. Tab 1: Transport; Tab 2: Restriction endonuclease; Tab 3: Glycolysis; Tab 4: Pyruvate oxidation; Tab 5: FtsH proteins; Tab 6: PMU core genes; Table S4: Singletons from the FDp genome.
