# Supplementary material for: The Complete Genome of the “Flavescence Dorée” Phytoplasma Reveals Characteristics of Low Genome Plasticity
Source: Biology (Basel). 2022 Jun 23;11(7):953. doi: 10.3390/biology11070953 (PMC9312162; doi:10.3390/biology11070953)

Supplementary Figure S1. Graphs from Phobius prediction of putative effectors

Prediction of FlDop_00023

ID	FlDop_00023			
FT	SIGNAL	1	32	
FT	REGION	1	12	N-REGION.
FT	REGION	13	24	H-REGION.
FT	REGION	25	32	C-REGION.
FT	TOPO_DOM	33	296	NON CYTOPLASMIC.
FT	TRANSMEM	297	316	
FT	TOPO_DOM	317	320	CYTOPLASMIC.
//				

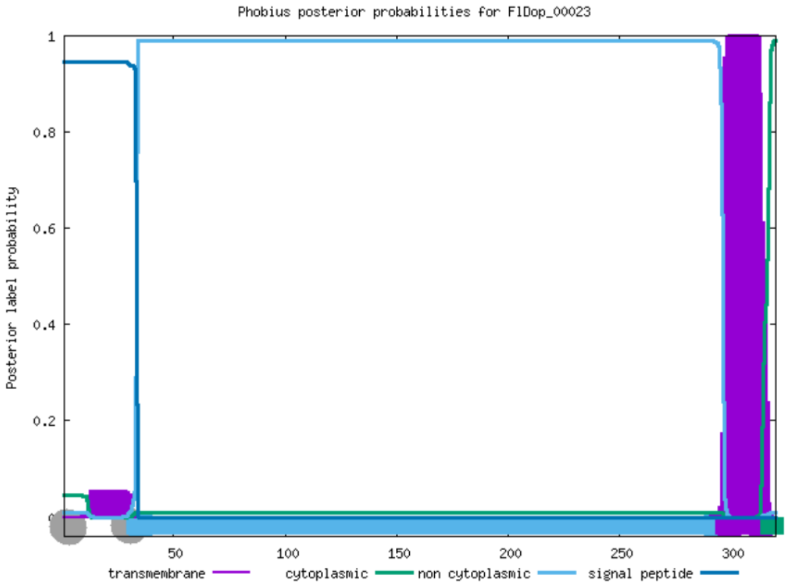

Prediction of FlDop_00035

ID	FlDop_00035			
FT	TOPO_DOM	1	11	CYTOPLASMIC.
FT	TRANSMEM	12	32	
FT	TOPO_DOM	33	497	NON CYTOPLASMIC.
//				

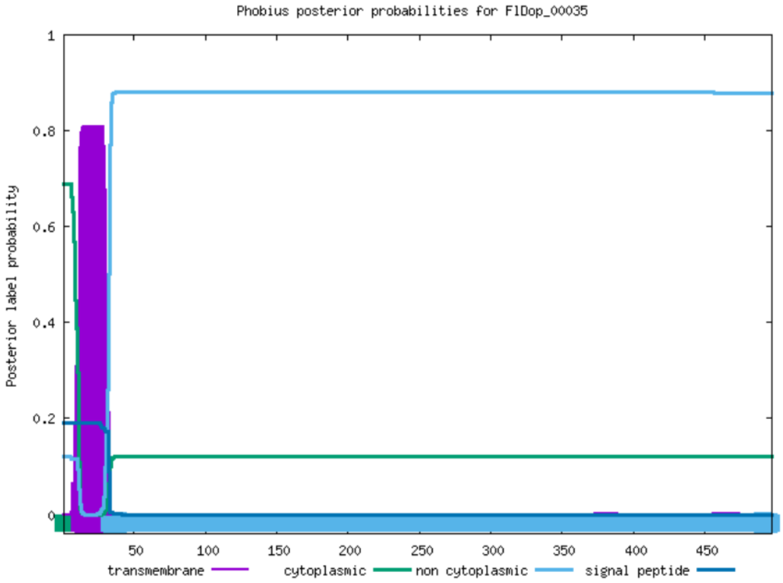

Prediction of FlDop_00041

```
ID   FlDop_00041
FT   TOPO_DOM      1     11       CYTOPLASMIC.
FT   TRANSMEM     12     34
FT   TOPO_DOM     35    228       NON CYTOPLASMIC.
FT   TRANSMEM    229    251
FT   TOPO_DOM    252    261       CYTOPLASMIC.
//
```

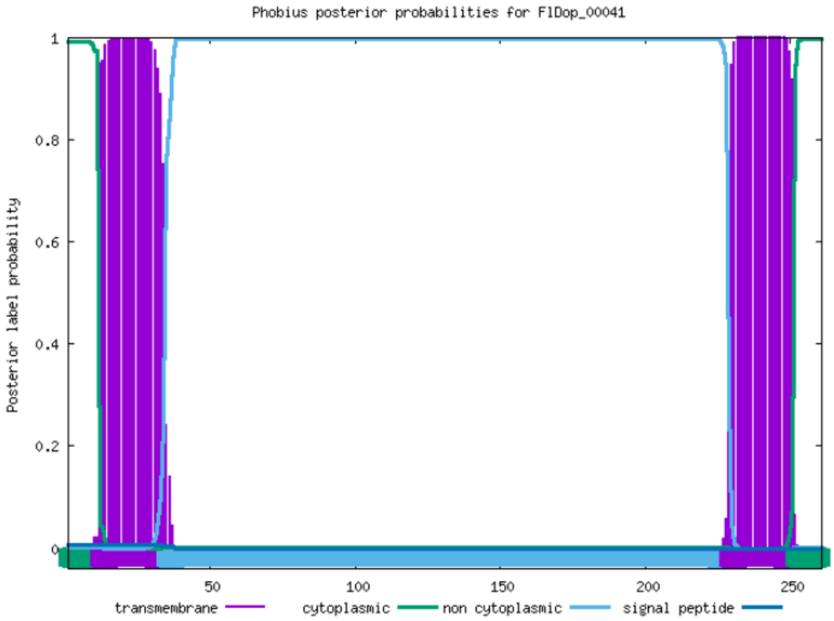

Prediction of FlDop_00048

```
ID   FlDop_00048
FT   SIGNAL        1     34
FT   REGION        1     14       N-REGION.
FT   REGION       15     30       H-REGION.
FT   REGION       31     34       C-REGION.
FT   TOPO_DOM     35    645       NON CYTOPLASMIC.
FT   TRANSMEM    646    666
FT   TOPO_DOM    667    673       CYTOPLASMIC.
//
```

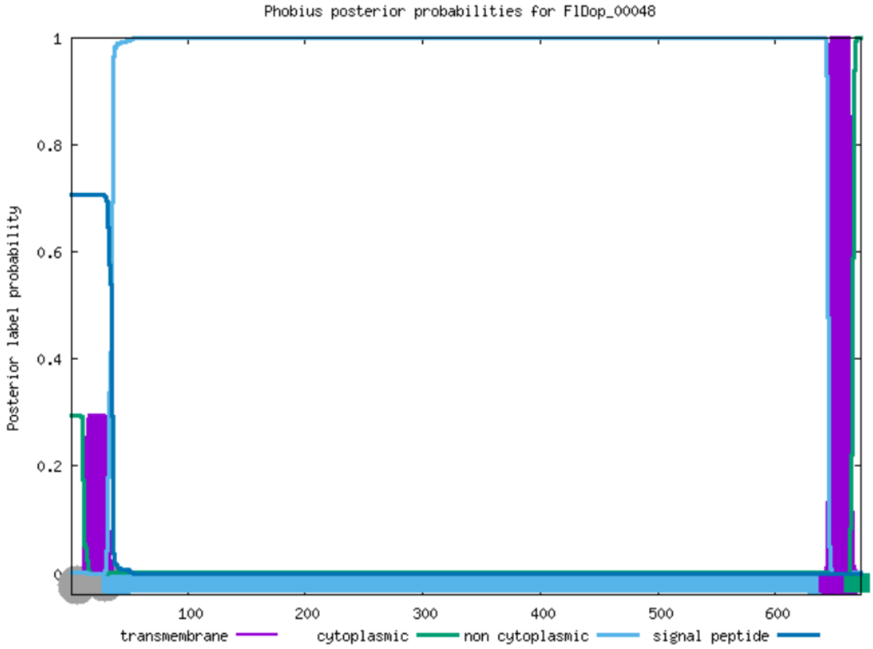

Prediction of FlDop_00049

```
ID    FlDop_00049
FT    TOPO_DOM      1     11       CYTOPLASMIC.
FT    TRANSMEM     12     32
FT    TOPO_DOM     33    248       NON CYTOPLASMIC.
FT    TRANSMEM    249    270
FT    TOPO_DOM    271    285       CYTOPLASMIC.
//
```

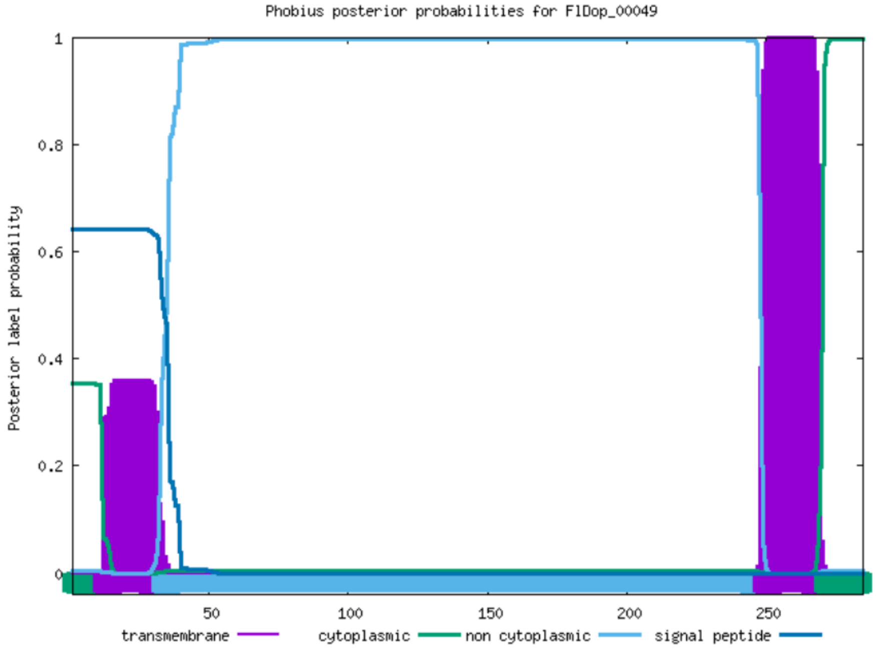

Prediction of FlDop_00090

```
ID    FlDop_00090
FT    SIGNAL        1     32
FT    REGION        1     12       N-REGION.
FT    REGION       13     24       H-REGION.
FT    REGION       25     32       C-REGION.
FT    TOPO_DOM     33    445       NON CYTOPLASMIC.
//
```

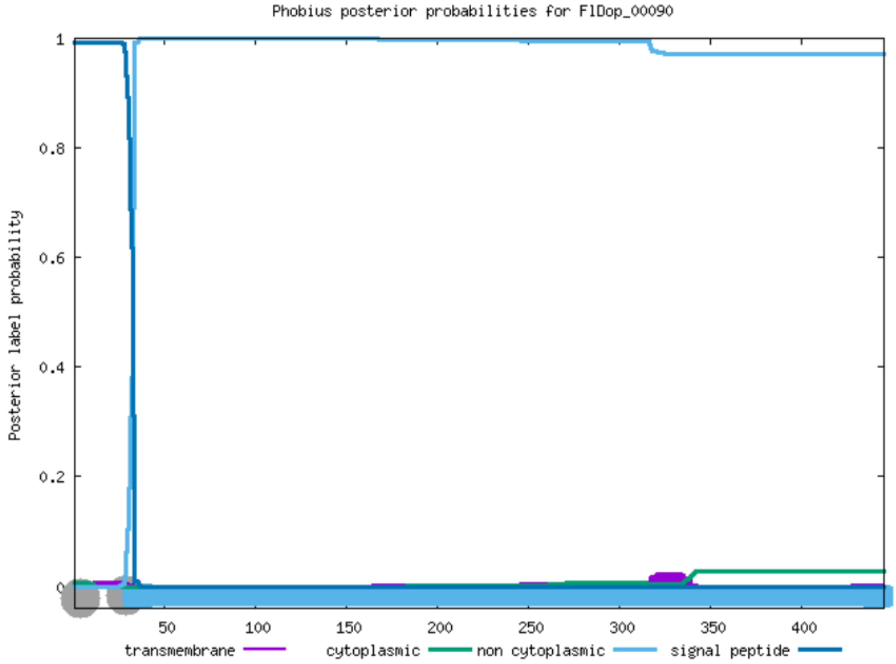

Prediction of FlDop_00101

```
ID   FlDop_00101
FT   TOPO_DOM      1     11       CYTOPLASMIC.
FT   TRANSMEM     12     32
FT   TOPO_DOM     33   1038       NON CYTOPLASMIC.
FT   TRANSMEM   1039   1058
FT   TOPO_DOM   1059   1059       CYTOPLASMIC.
//
```

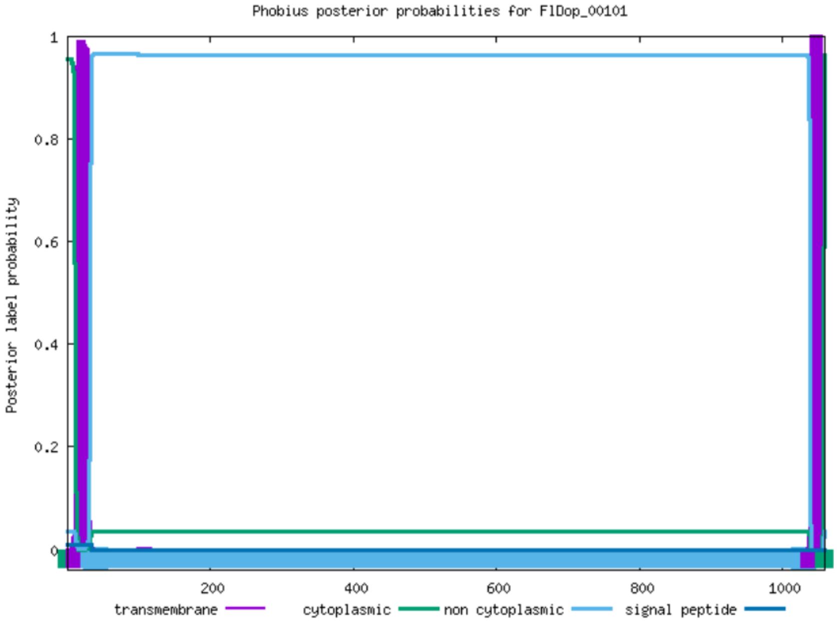

Prediction of FlDop_00112

```
ID   FlDop_00112
FT   SIGNAL        1     19
FT   REGION        1      2       N-REGION.
FT   REGION        3     14       H-REGION.
FT   REGION       15     19       C-REGION.
FT   TOPO_DOM     20    108       NON CYTOPLASMIC.
//
```

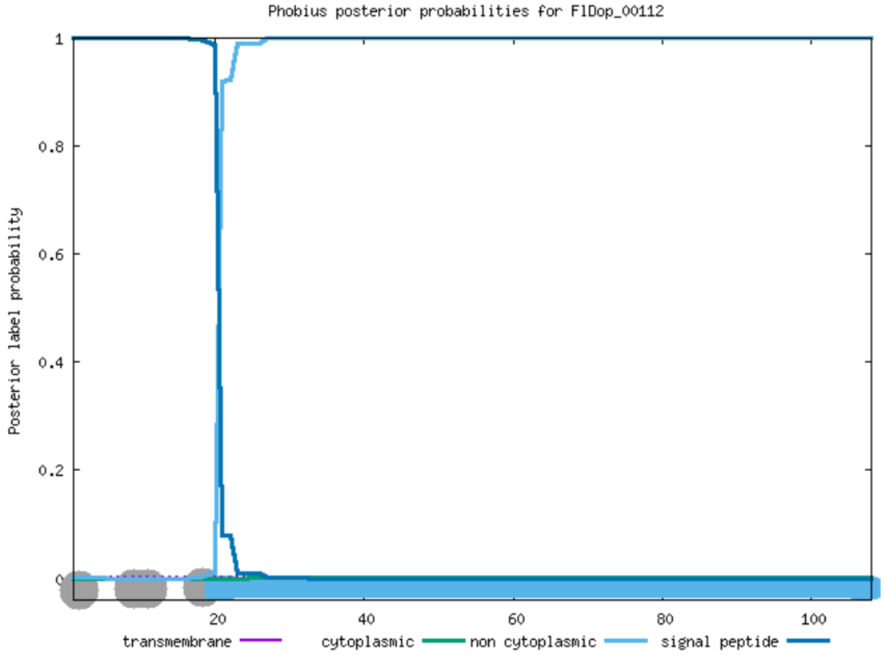

Prediction of FlDop_00153

```
ID   FlDop_00153
FT   TOPO_DOM      1     13       NON CYTOPLASMIC.
FT   TRANSMEM     14     35
FT   TOPO_DOM     36    382       CYTOPLASMIC.
//
```

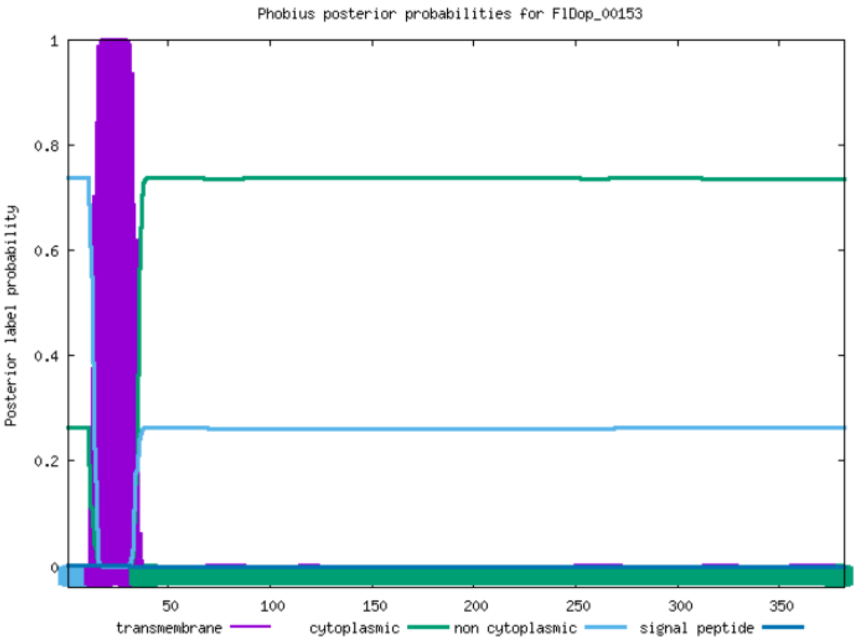

Prediction of FlDop_00158

```
ID   FlDop_00158
FT   SIGNAL        1     36
FT   REGION        1     15       N-REGION.
FT   REGION       16     28       H-REGION.
FT   REGION       29     36       C-REGION.
FT   TOPO_DOM     37    234       NON CYTOPLASMIC.
//
```

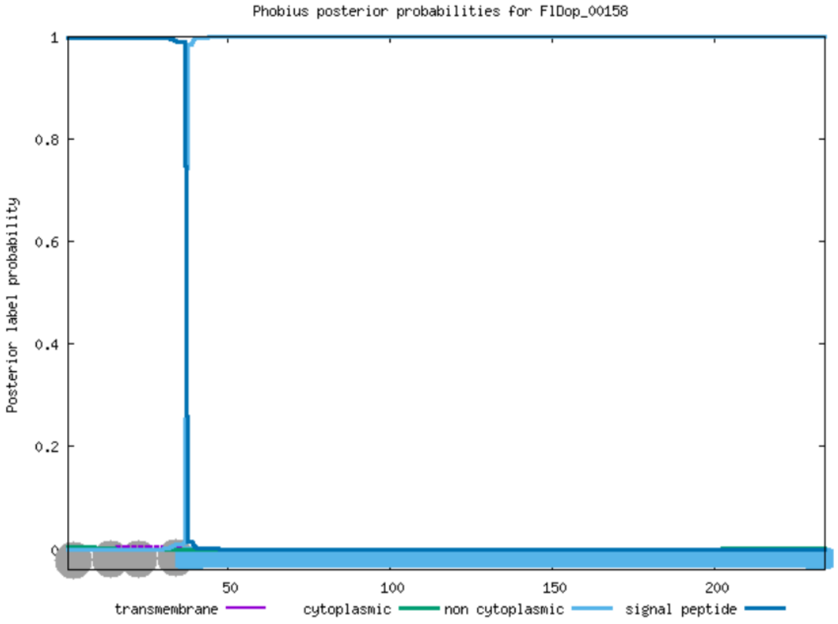

Prediction of FlDop_00183

```
ID   FlDop_00183
FT   SIGNAL        1     33
FT   REGION        1     12        N-REGION.
FT   REGION       13     24        H-REGION.
FT   REGION       25     33        C-REGION.
FT   TOPO_DOM     34    137        NON CYTOPLASMIC.
//
```

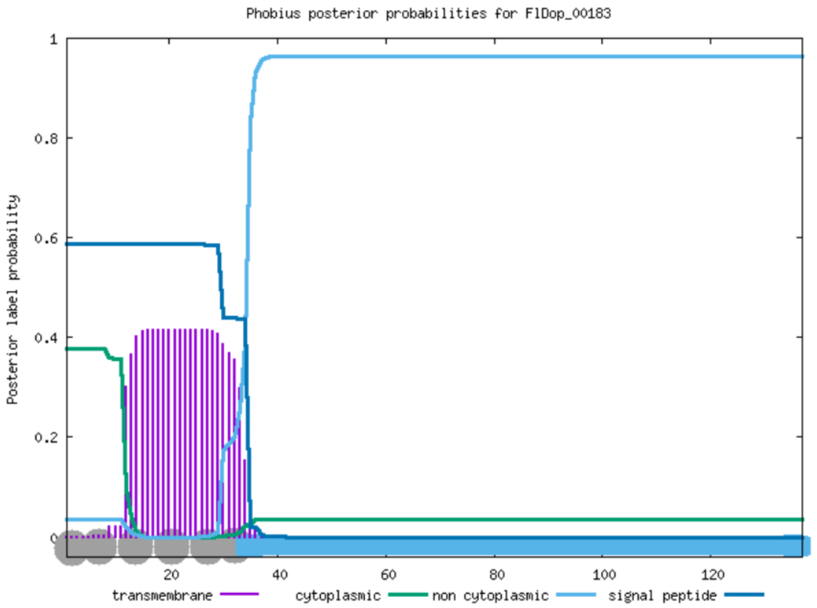

Prediction of FlDop_00185

```
ID   FlDop_00185
FT   SIGNAL        1     32
FT   REGION        1      9        N-REGION.
FT   REGION       10     25        H-REGION.
FT   REGION       26     32        C-REGION.
FT   TOPO_DOM     33    125        NON CYTOPLASMIC.
//
```

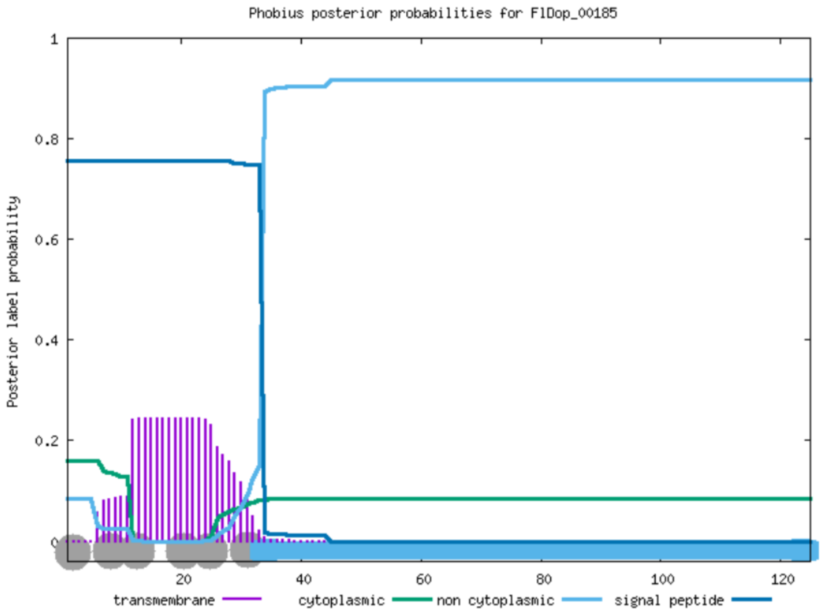

Prediction of FlDop_00190

```
ID   FlDop_00190
FT   SIGNAL        1     17
FT   REGION        1      4       N-REGION.
FT   REGION        5     12       H-REGION.
FT   REGION       13     17       C-REGION.
FT   TOPO_DOM     18    117       NON CYTOPLASMIC.
//
```

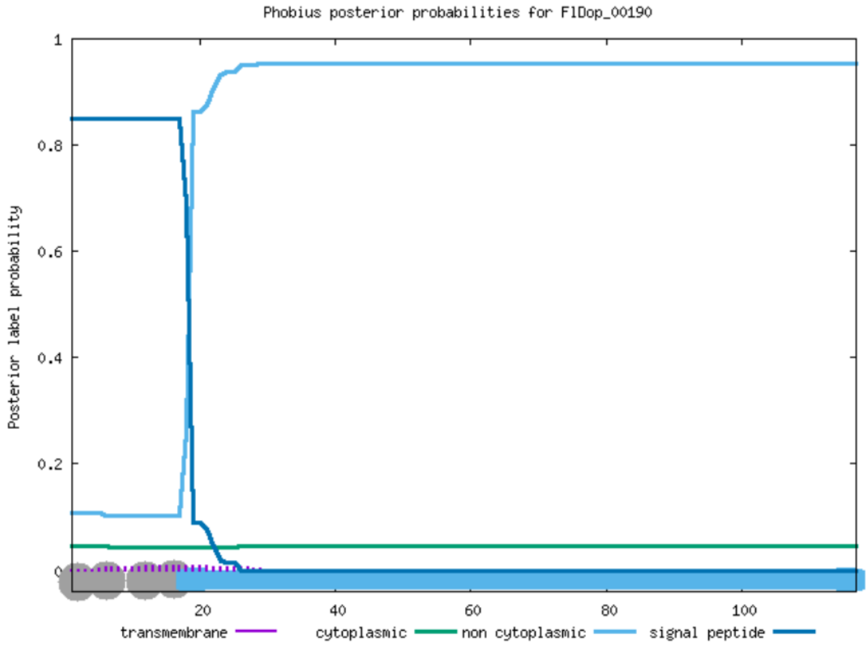

Prediction of FlDop_00246

```
ID   FlDop_00246
FT   SIGNAL        1     30
FT   REGION        1     13       N-REGION.
FT   REGION       14     25       H-REGION.
FT   REGION       26     30       C-REGION.
FT   TOPO_DOM     31    211       NON CYTOPLASMIC.
//
```

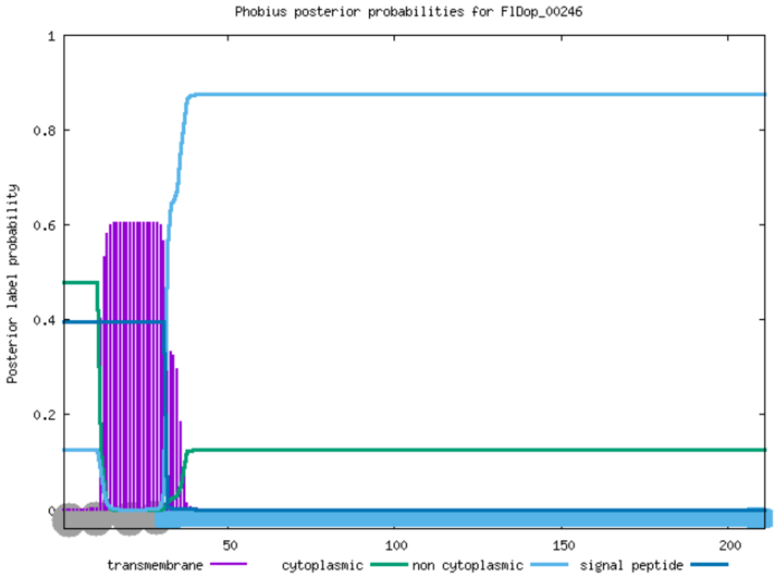

Prediction of FlDop_00265

```
ID   FlDop_00265
FT   TOPO_DOM      1      6       CYTOPLASMIC.
FT   TRANSMEM      7     27
FT   TOPO_DOM     28    626       NON CYTOPLASMIC.
//
```

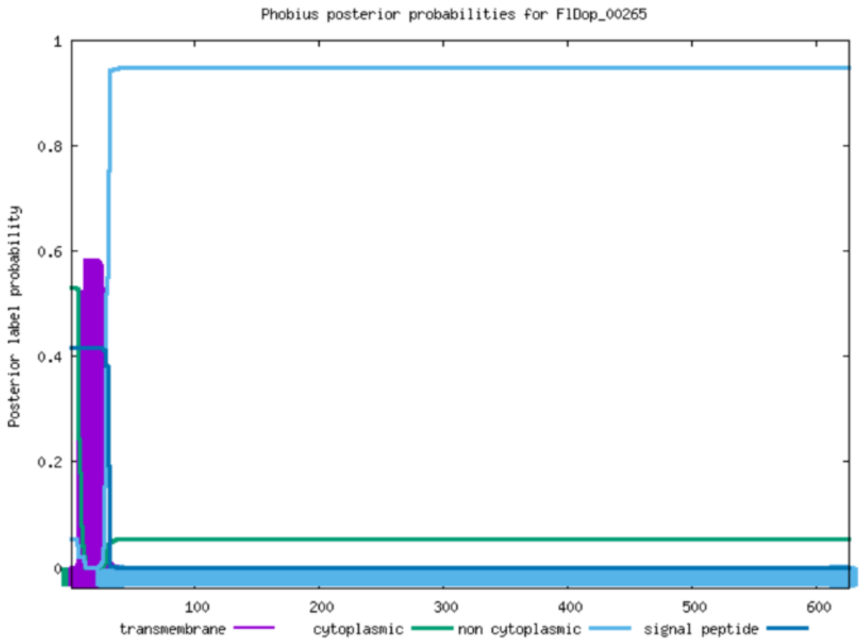

Prediction of FlDop_00266

```
ID   FlDop_00266
FT   SIGNAL        1     35
FT   REGION        1     18       N-REGION.
FT   REGION       19     30       H-REGION.
FT   REGION       31     35       C-REGION.
FT   TOPO_DOM     36    450       NON CYTOPLASMIC.
//
```

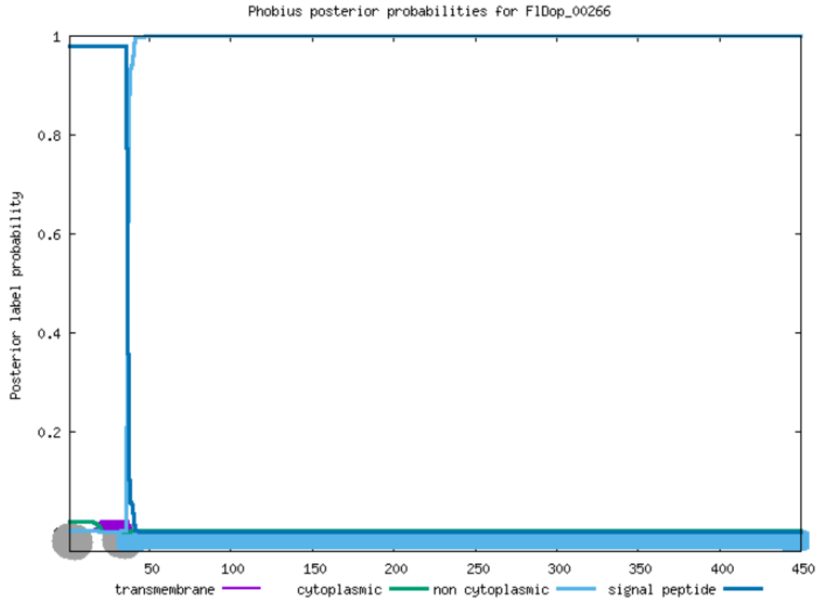

Prediction of FlDop_00269

```
ID   FlDop_00269
FT   TOPO_DOM      1     11       CYTOPLASMIC.
FT   TRANSMEM     12     32
FT   TOPO_DOM     33    497       NON CYTOPLASMIC.
//
```

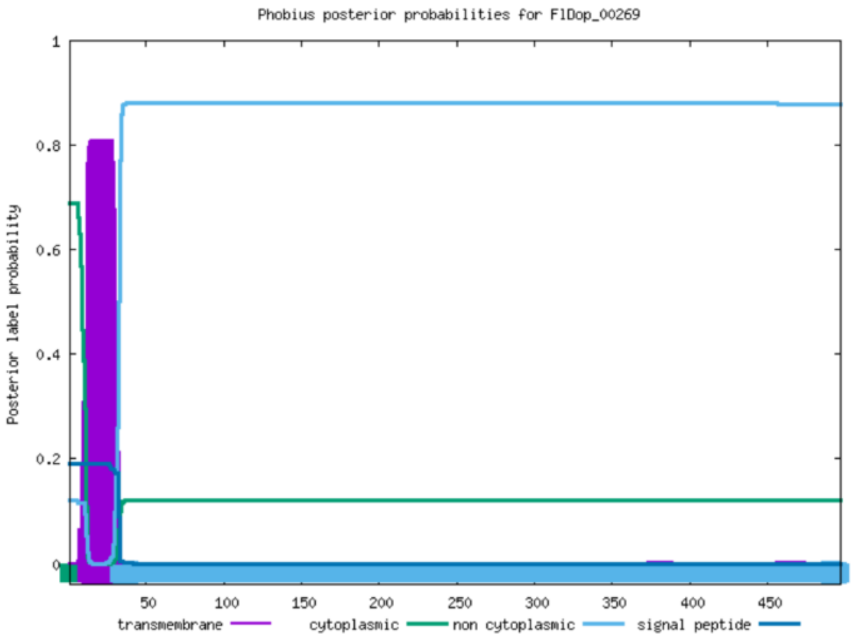


```
ID   FlDop_00278
FT   SIGNAL        1     16
FT   REGION        1      1       N-REGION.
FT   REGION        2     10       H-REGION.
FT   REGION       11     16       C-REGION.
FT   TOPO_DOM     17    118       NON CYTOPLASMIC.
//
```

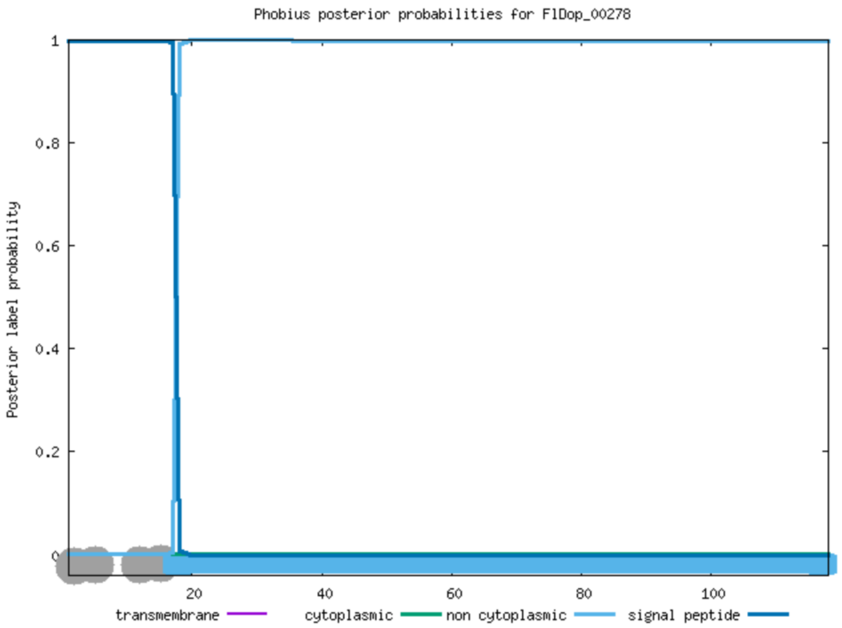

Prediction of FlDop_00279

```
ID    FlDop_00279
FT    TOPO_DOM       1     11       CYTOPLASMIC.
FT    TRANSMEM      12     31
FT    TOPO_DOM      32     83       NON CYTOPLASMIC.
FT    TRANSMEM      84    104
FT    TOPO_DOM     105    131       CYTOPLASMIC.
//
```

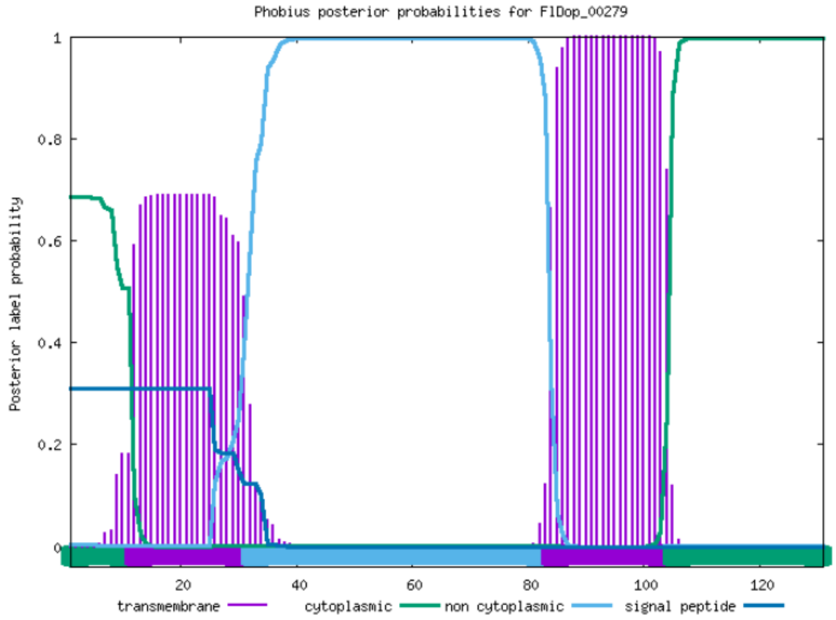

ftsH protein

```
ID    FlDop_00292
FT    TOPO_DOM       1     11       CYTOPLASMIC.
FT    TRANSMEM      12     32
FT    TOPO_DOM      33    125       NON CYTOPLASMIC.
FT    TRANSMEM     126    156
FT    TOPO_DOM     157    599       CYTOPLASMIC.
//
```

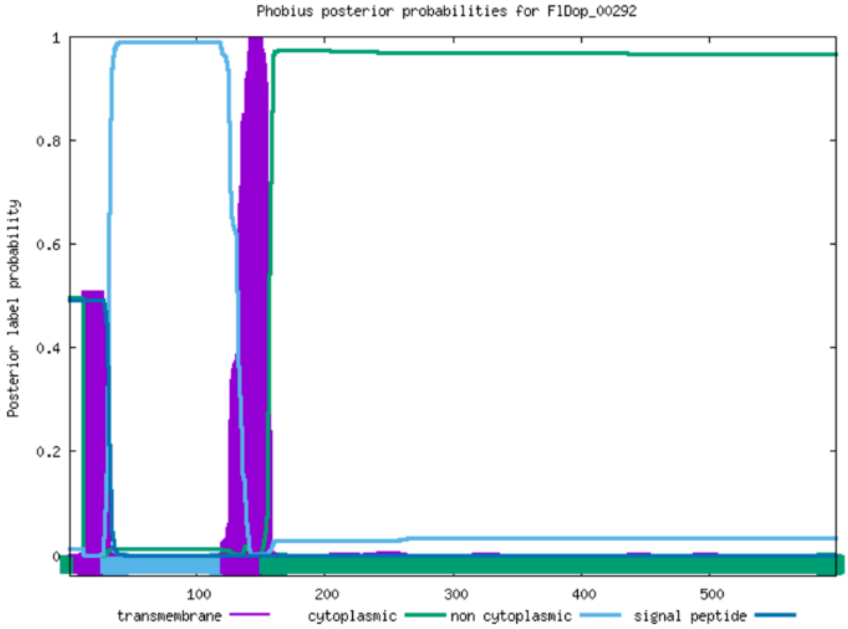

```
ID   FlDop_00298
FT   SIGNAL         1     33
FT   REGION         1     12       N-REGION.
FT   REGION        13     24       H-REGION.
FT   REGION        25     33       C-REGION.
FT   TOPO_DOM      34    398       NON CYTOPLASMIC.
//
```

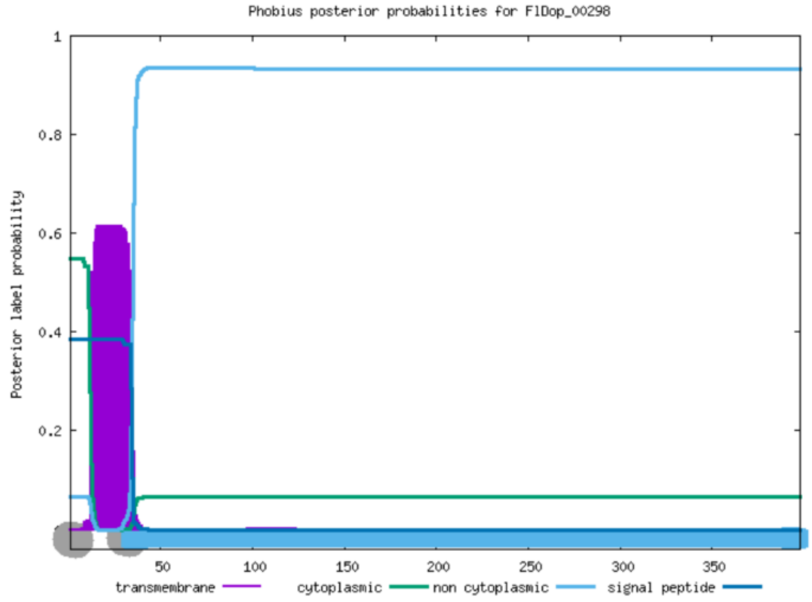

Prediction of FlDop_00402

```
ID   FlDop_00402
FT   TOPO_DOM       1     11       CYTOPLASMIC.
FT   TRANSMEM      12     39
FT   TOPO_DOM      40    170       NON CYTOPLASMIC.
FT   TRANSMEM     171    189
FT   TOPO_DOM     190    204       CYTOPLASMIC.
//
```

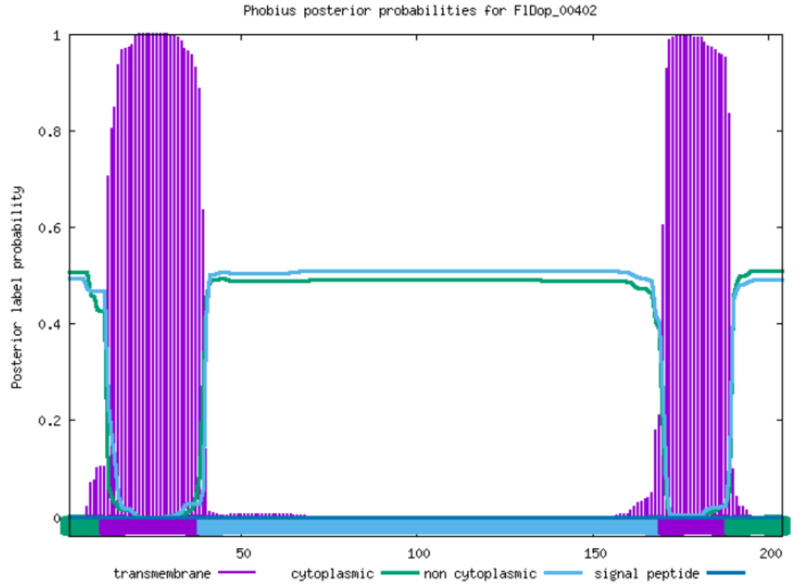

```
ID   FlDop_00420
FT   SIGNAL         1     29
FT   REGION         1     13       N-REGION.
FT   REGION        14     25       H-REGION.
FT   REGION        26     29       C-REGION.
FT   TOPO_DOM      30    324       NON CYTOPLASMIC.
//
```

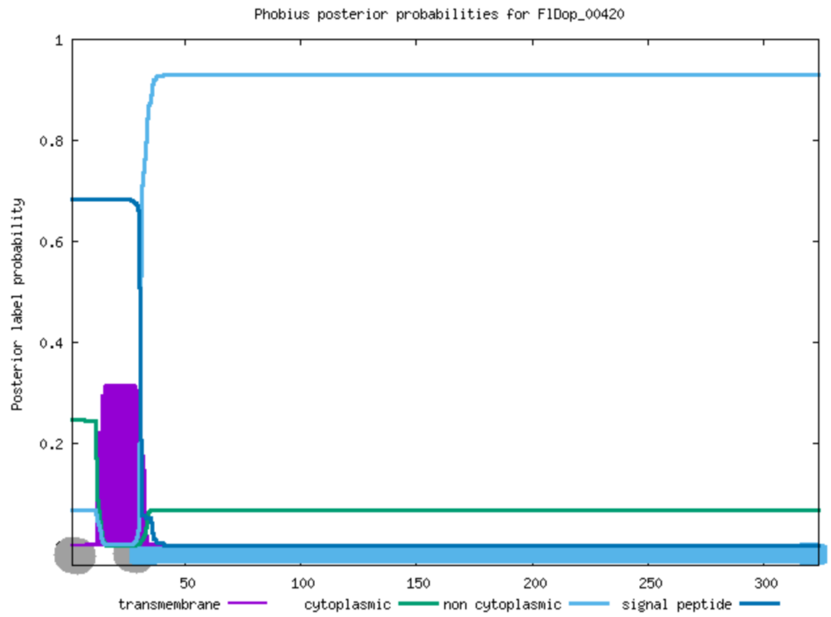

Prediction of FlDop_00531

```
ID   FlDop_00531
FT   SIGNAL         1     32
FT   REGION         1     12       N-REGION.
FT   REGION        13     24       H-REGION.
FT   REGION        25     32       C-REGION.
FT   TOPO_DOM      33    122       NON CYTOPLASMIC.
//
```

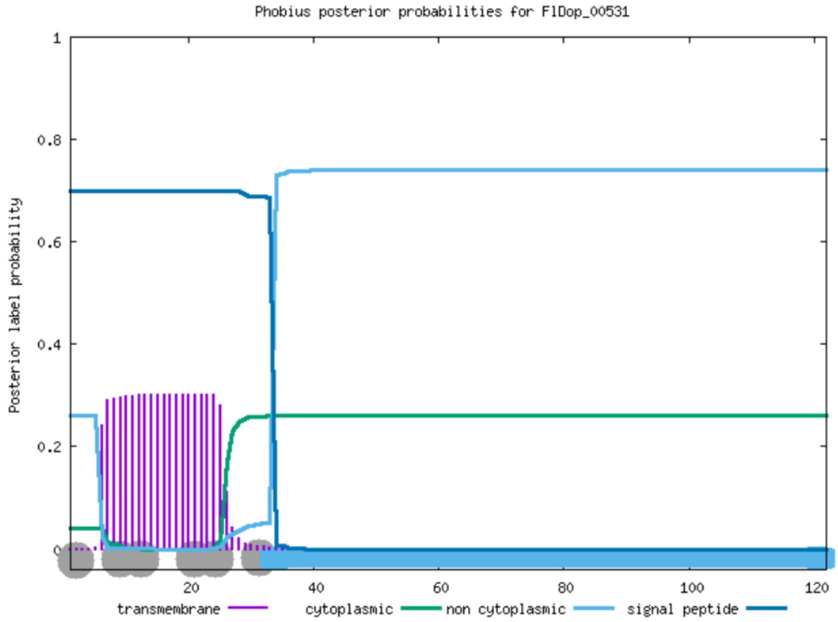

Supplement: Supplementary file 1 [file biology-11-00953-s001.zip › supplementary_Figure S1.pdf]
