# Supplementary material for: The Complete Genome of the “Flavescence Dorée” Phytoplasma Reveals Characteristics of Low Genome Plasticity
Source: Biology (Basel). 2022 Jun 23;11(7):953. doi: 10.3390/biology11070953 (PMC9312162; doi:10.3390/biology11070953)

Figure S2. GC skew plots generated with GenSkew online tool (https://genskew.csb.univie.ac.at/webskew). Plots are displayed with a sliding window of 5 kb and a step size of 0.5 kb.

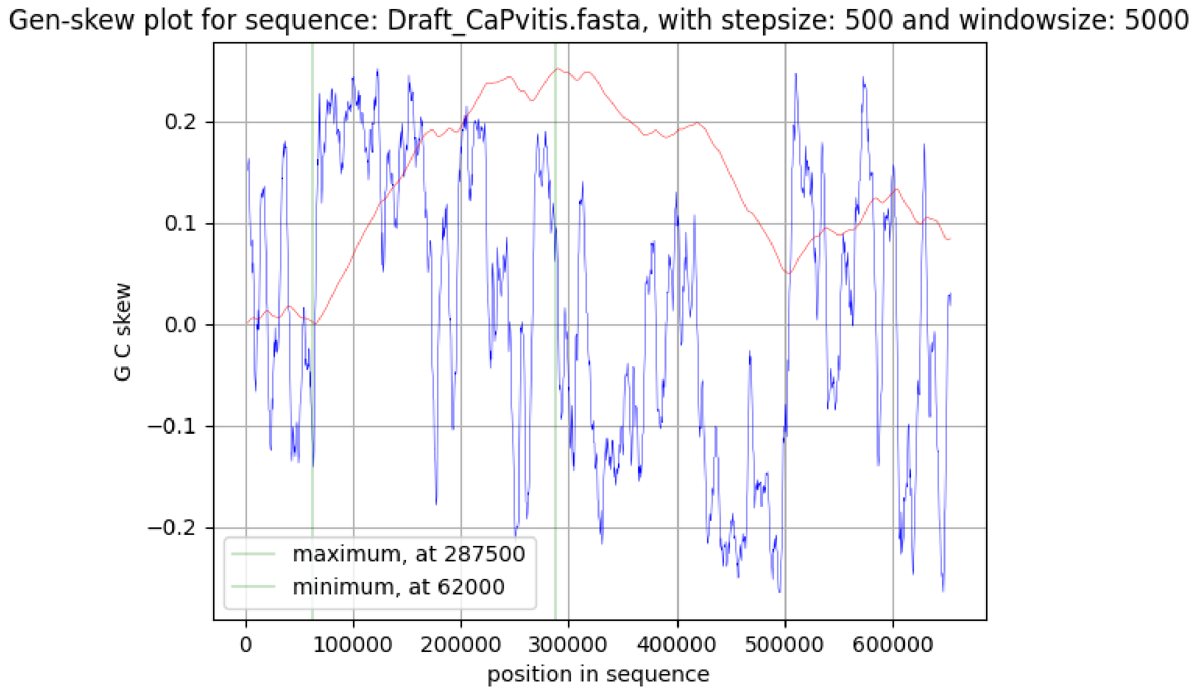

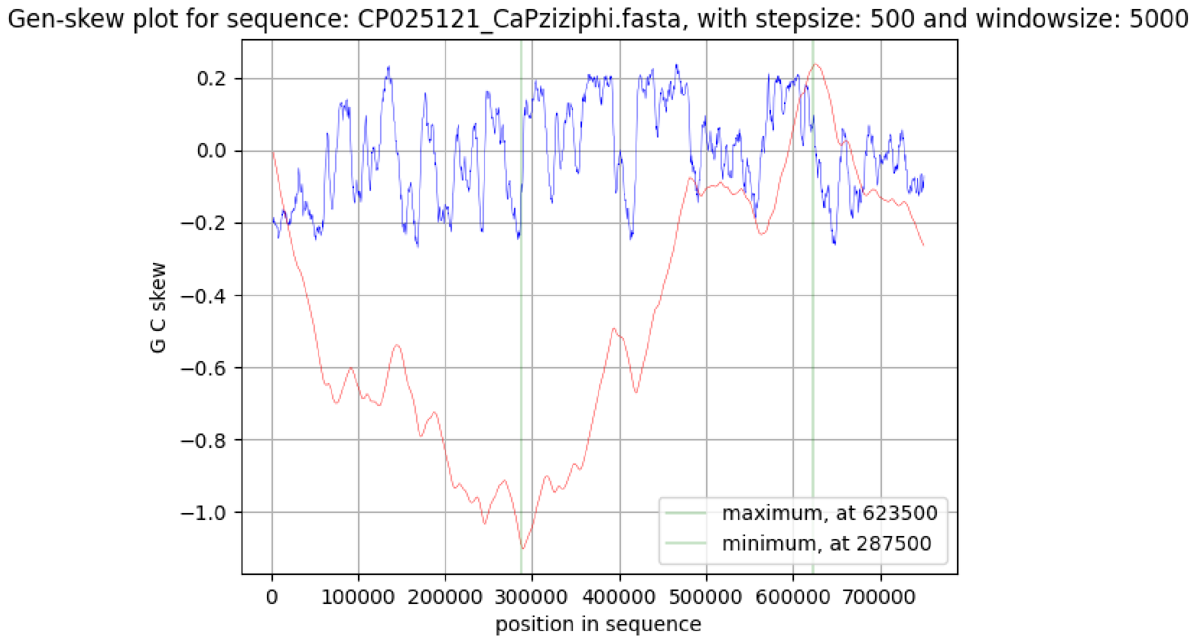

Gen-skew plot for sequence: NZ_CP054393_CaPluffae.fasta, with stepsize: 500 and windowsize: 5000

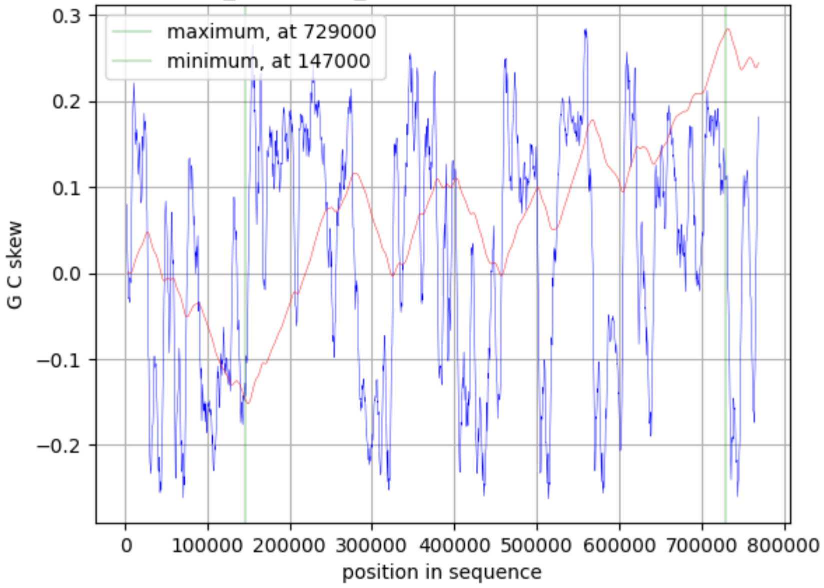

Gen-skew plot for sequence: NC_011047_CaPmali.fasta, with stepsize: 500 and windowsize: 5000

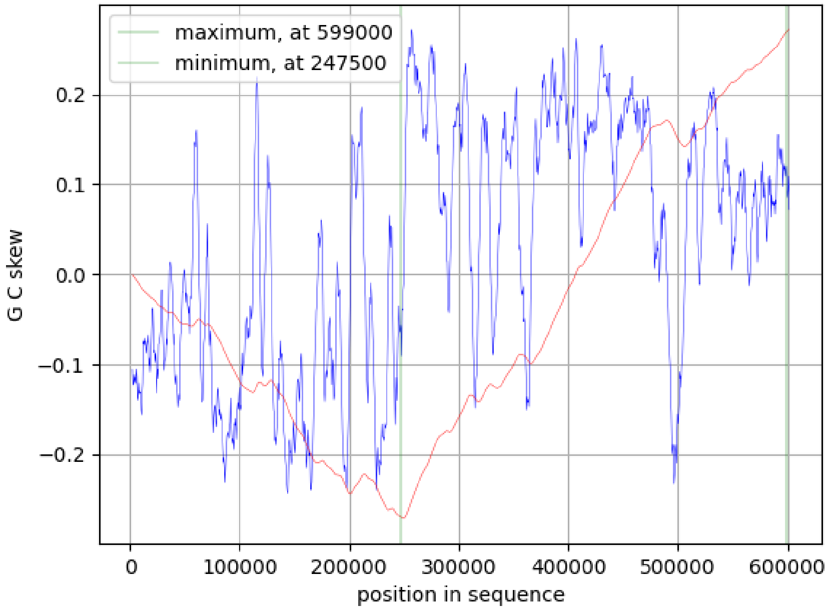

Gen-skew plot for sequence: CP040925_CaPaurantifolia.fasta, with stepsize: 500 and windowsize: 5000

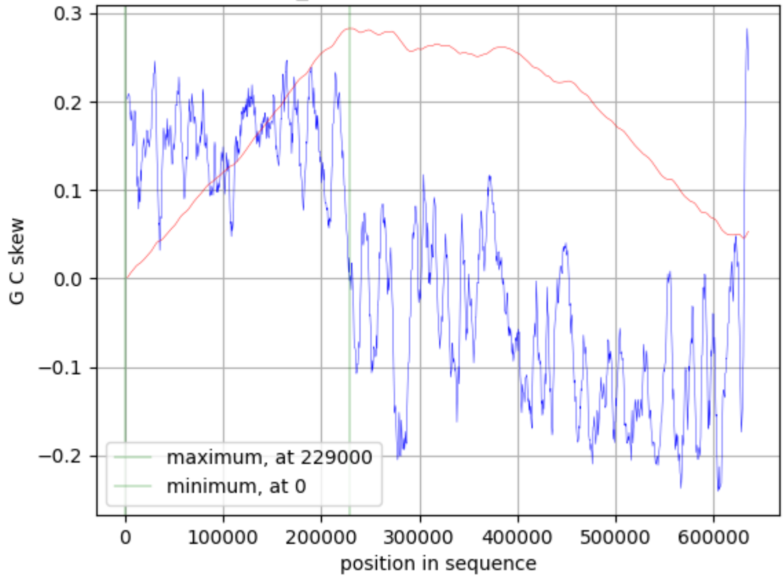

Gen-skew plot for sequence: AP006628_CaPasteris_OYM.fasta, with stepsize: 500 and windowsize: 5000

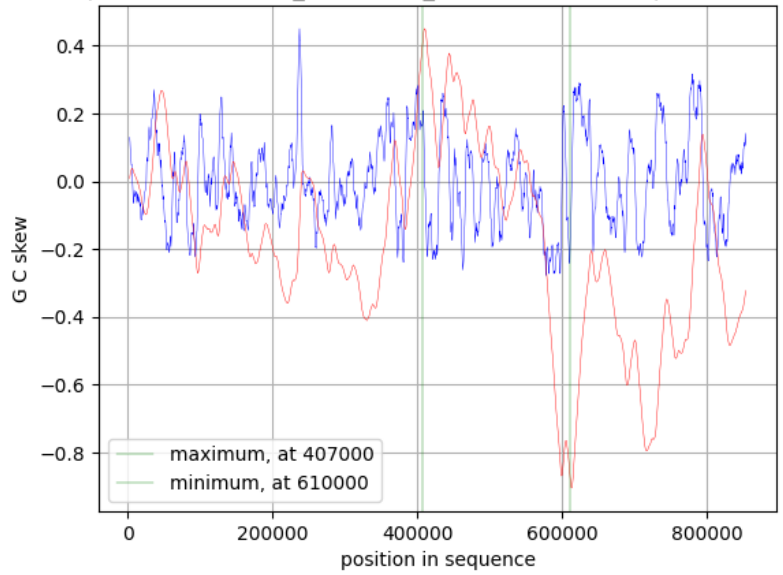

Gen-skew plot for sequence: NC_007716_CaPasteris_AYWB.fasta, with stepsize: 500 and windowsize: 5000

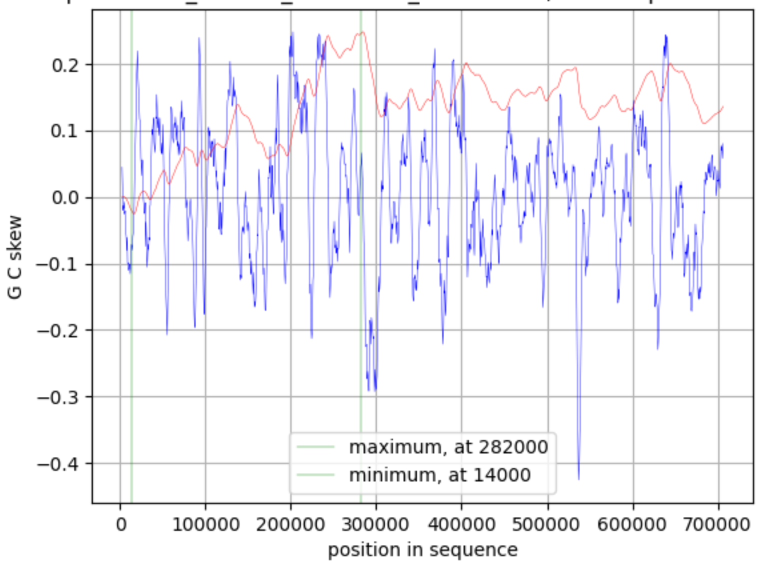

Gen-skew plot for sequence: NC_021236_CaPaustraliense.fasta, with stepsize: 500 and windowsize: 5000

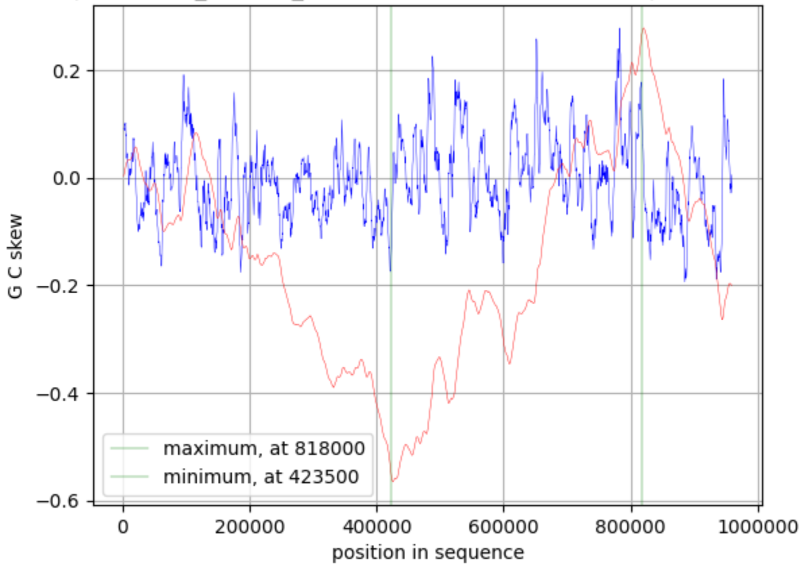

Supplement: Supplementary file 1 [file biology-11-00953-s001.zip › supplementary_Figure S2.pdf]
